# Impact of a TAK-1 inhibitor as a single or as an add-on therapy to riociguat on the metabolic reprograming and pulmonary hypertension in the SUGEN5416/hypoxia rat model

**DOI:** 10.3389/fphar.2023.1021535

**Published:** 2023-03-29

**Authors:** Daniel Morales-Cano, Jose Luis Izquierdo-García, Bianca Barreira, Sergio Esquivel-Ruiz, Maria Callejo, Rachele Pandolfi, Palmira Villa-Valverde, Ignacio Rodríguez, Angel Cogolludo, Jesus Ruiz-Cabello, Francisco Perez-Vizcaino, Laura Moreno

**Affiliations:** ^1^ Department of Pharmacology and Toxicology, School of Medicine, Universidad Complutense de Madrid, Madrid, Spain; ^2^ Ciber Enfermedades Respiratorias (Ciberes), Madrid, Spain; ^3^ Instituto de Investigación Sanitaria Gregorio Marañón (IISGM), Madrid, Spain; ^4^ Centro Nacional de Investigaciones Cardiovasculares (CNIC), Madrid, Spain; ^5^ Department of Clinical Medicine, Aarhus University, Aarhus, Denmark; ^6^ Department of Chemistry in Pharmaceutical Sciences, School of Pharmacy, Universidad Complutense de Madrid, Madrid, Spain; ^7^ Instituto Pluridisciplinar, Universidad Complutense de Madrid, Madrid, Spain; ^8^ ICTS Bioimagen Complutense, Universidad Complutense de Madrid, Madrid, Spain; ^9^ Center for Cooperative Research in Biomaterials (CIC biomaGUNE), Basque Research and Technology Alliance (BRTA), Donostia San Sebastián, Spain

**Keywords:** pulmonary hypertension, antiproliferative, metabolomics, combination therapy, right ventricle

## Abstract

**Background:** Despite increasing evidence suggesting that pulmonary arterial hypertension (PAH) is a complex disease involving vasoconstriction, thrombosis, inflammation, metabolic dysregulation and vascular proliferation, all the drugs approved for PAH mainly act as vasodilating agents. Since excessive TGF-β signaling is believed to be a critical factor in pulmonary vascular remodeling, we hypothesized that blocking TGFβ-activated kinase 1 (TAK-1), alone or in combination with a vasodilator therapy (i.e., riociguat) could achieve a greater therapeutic benefit.

**Methods:** PAH was induced in male Wistar rats by a single injection of the VEGF receptor antagonist SU5416 (20 mg/kg) followed by exposure to hypoxia (10%O_2_) for 21 days. Two weeks after SU5416 administration, vehicle, riociguat (3 mg/kg/day), the TAK-1 inhibitor 5Z-7-oxozeaenol (OXO, 3 mg/kg/day), or both drugs combined were administered for 7 days. Metabolic profiling of right ventricle (RV), lung tissues and PA smooth muscle cells (PASMCs) extracts were performed by magnetic resonance spectroscopy, and the differences between groups analyzed by multivariate statistical methods.

**Results:** *In vitro*, riociguat induced potent vasodilator effects in isolated pulmonary arteries (PA) with negligible antiproliferative effects and metabolic changes in PASMCs. In contrast, 5Z-7-oxozeaenol effectively inhibited the proliferation of PASMCs characterized by a broad metabolic reprogramming but had no acute vasodilator effects. *In vivo,* treatment with riociguat partially reduced the increase in pulmonary arterial pressure (PAP), RV hypertrophy (RVH), and pulmonary vascular remodeling, attenuated the dysregulation of inosine, glucose, creatine and phosphocholine (PC) in RV and fully abolished the increase in lung IL-1β expression. By contrast, 5Z-7-oxozeaenol significantly reduced pulmonary vascular remodeling and attenuated the metabolic shifts of glucose and PC in RV but had no effects on PAP or RVH. Importantly, combined therapy had an additive effect on pulmonary vascular remodeling and induced a significant metabolic effect over taurine, amino acids, glycolysis, and TCA cycle metabolism *via* glycine-serine-threonine metabolism. However, it did not improve the effects induced by riociguat alone on pulmonary pressure or RV remodeling. None of the treatments attenuated pulmonary endothelial dysfunction and hyperresponsiveness to serotonin in isolated PA.

**Conclusion:** Our results suggest that inhibition of TAK-1 induces antiproliferative effects and its addition to short-term vasodilator therapy enhances the beneficial effects on pulmonary vascular remodeling and RV metabolic reprogramming in experimental PAH.

## Introduction

Pulmonary arterial hypertension (PAH) is a chronic and progressive disease characterized by a sustained elevation of pulmonary arterial pressure (PAP) and associated with high morbidity and mortality (Simonneau et al., 2013; Nicolls and Voelkel, 2017; Humbert et al., 2019). As disease severity progresses, right ventricular (RV) function worsens due to sustained pressure overload, leading to RV failure. Indeed, RV failure remains the primary cause of death in patients with PAH (Vonk Noordegraaf et al., 2017).

The pathophysiology of PAH is complex, involving vasoconstriction, thrombosis, inflammation, metabolic dysregulation and vascular proliferation. While much progress has been made in the treatment of PAH, currently approved drugs for PAH act as primary vasodilating agents targeting endothelin, nitric oxide, and prostacyclin pathways (Rabinovitch, 2012; Nicolls and Voelkel, 2017; Prins et al., 2019a; Humbert et al., 2019; Koudstaal et al., 2020). Although these therapies have led to significant improvements in overall medial survival and quality of life of patients, none of them are curative, and PAH remains a fatal disease (Montani et al., 2014; Scott et al., 2021). Furthermore, despite RV dysfunction determines the prognosis of PAH patients, therapies targeting the RV are still needed (Vonk Noordegraaf et al., 2017). Therefore, novel therapies must go beyond vasodilatation and start targeting maladaptive vascular and RV remodeling if we aim to improve the outcomes in PAH patients and progress toward a cure (Prins et al., 2019a; Prins et al., 2019b; Scott et al., 2021).

Extensive research during the last decades has identified new cellular and molecular pathways that have yet to be exploited therapeutically (Prins et al., 2019b; Andre et al., 2021; Scott et al., 2021; Sharmin et al., 2021). For example, dysregulation of the TGF-β superfamily pathways has been proven critical in the pathogenesis of PAH (Rol et al., 2018; Andre et al., 2021; Sharmin et al., 2021). Thus, in PAH, deficient bone morphogenetic protein (BMP) type II receptor (BMPR-II) impairs the anti-proliferative SMAD 1/5/8 signaling pathways whilst the overactive TGFβ pathway leads to activation of the SMAD2/3 pathway signaling and results in excessive proliferation, attenuation of apoptosis, production of inflammatory cytokines, mitochondrial dysfunction (Marsboom et al., 2012; Freund-Michel et al., 2014) and a metabolic reprogramming mainly characterized by a shift in glucose metabolism from oxidative phosphorylation to glycolysis (also known as the Warburg effect (Rol et al., 2018; Hernandez-Saavedra et al., 2020; Scott et al., 2021; Sharmin et al., 2021). These pieces of evidence suggest that therapeutic interventions eliciting anti-TGF-β and pro-BMP effects have the potential to be disease modifying. Indeed, sotatercept, a ligand trap for activin (another member of the TGF-β superfamily) which is able to restore the balance between BMP/TGF-β is currently undergoing a Phase III clinical trial following the encouraging results from the Phase II PULSAR trial (Humbert et al., 2021).

In this regard, TGF-β-associated kinase 1 (TAK1) seems crucial in the cross-regulation between the BMP and the TGFβ arms. Thus, with fully functional BMPR-II-mediated signaling, TAK1 is retained by the BMPR-II receptor complex. By contrast, through BMPR2 dysregulation, the TAK1 intracellular pool becomes available for the TGFβ receptor complex, leading to abnormal proliferation and apoptosis (Nasim et al., 2012). In line with this, TAK1 has been shown to mediate myocardial and vascular remodeling in systemic hypertension (Song et al., 2014; Garfield et al., 2019; Meijles et al., 2020).

Based on these evidences, we hypothesized that sustained inhibition of TAK1 with the low molecular weight inhibitor 5Z-7-oxozeaenol in combination with a vasodilator therapy such as the soluble guanylate cyclase (sGC) stimulator riociguat could ameliorate or even reverse pulmonary vascular remodeling and RV dysfunction in PAH by their ability to limit inflammation and metabolic disruption and to mitigate vascular and cardiac remodeling. Pharmacological treatment was evaluated in pulmonary arterial smooth muscle cells (PASMCs) and a relevant PAH animal model to validate our hypothesis. Finally, cell extract and intact lung and heart tissues were analyzed by Nuclear Magnetic Resonance spectroscopy (NMR) to characterize the metabolic state after treatment.

## Materials and methods

All experimental procedures involving animals were approved by the Institutional and Regional Ethical Committees of Madrid (Spain; PROEX 251/15). Male Wistar rats (8–10 weeks of age; body weight ≈220 g) obtained from Envigo (Barcelona, Spain) were kept in the Animal Core Facility of University Complutense of Madrid under standard conditions of temperature (24°C ± 1°C) and 12 h dark/light cycle with *ad libitum* access to food and water. Animal studies are reported in compliance with the ARRIVE guidelines (Kilkenny et al., 2010).

### Experimental procedure for the rat SU5416/Hypoxia model

Adult male Wistar rats were injected subcutaneously with a single dose of the VEGFR type 2 inhibitor SUGEN 5416 (SU5416; 20 mg/kg) or the equivalent volume of the vehicle CMC (0.5% [w/v] carboxymethyl cellulose sodium, 0.9% [w/v] sodium chloride, 0.4% [v/v] Tween 80, 0.9% [v/v] benzyl alcohol in deionized water) before being exposed to normobaric hypoxia (10% oxygen) for 3 weeks (SuHyp model) (Moral-Sanz et al., 2012; Mondejar-Parreno et al., 2019; Callejo et al., 2020). 2 weeks after the administration of SU5416, rats were further randomly assigned to receive the treatment with vehicle, the TAK-1 inhibitor 5Z-7-oxozeaenol (3 mg/kg/day), riociguat (3 mg/kg/day) or both drugs combined for an additional period of 7 days. Drugs were administered by daily intraperitoneal injection following the study flowchart shown in Supplementary Figure S1A.

### Hemodynamic measurements

Twenty-four hours after the last administration of each treatment, the rats were anesthetized (80 mg/kg ketamine and 8 mg/kg xylazine i. p.), intubated through a tracheostomy and ventilated with room air (tidal volume 9 ml/kg, 60 breaths/min, and a positive end-expiratory pressure of 2 cm H_2_O, Nemi Scientific Inc., Medway, United States of America (Cogolludo et al., 2009; Moral-Sanz et al., 2012; Morales-Cano et al., 2014). Using an open-chest approach, a pressure transducer was placed, *via* a catheter, into the RV and then advanced into the pulmonary artery (PA). Systolic, end-diastolic and mean RV pressure (RVSP, RVEDP and RVMP) and systolic, diastolic and mean PA pressures (SPAP, DPAP and MPAP) were measured over three consecutive stable recordings. It should be noted that the pressure and heart rate values obtained in anaesthetized open-chest rats may represent an underestimation of the values in closed-chest conscious animals (Cogolludo et al., 2009; Moral-Sanz et al., 2012; Morales-Cano et al., 2014).

### Assessment of RV hypertrophy

At the end of the recordings, animals were sacrificed by overdose of anesthesia. Hearts were excised and the RV and the left ventricle plus septum (LV + S) were carefully dissected and weighed before being snap frozen for subsequent analysis. The ratios RV to body weight and RV to LV + S (Fulton Index) were calculated to assess the RV hypertrophy.

#### Lung histology

The left lung lobes were immediately collected for vascular reactivity or snap frozen for metabolomic profiling. The right lung was inflated *in situ* with formol saline through the right bronchus and embedded in paraffin. Lung sections were stained with eosin and hematoxylin, and elastin was visualized by its green autofluorescence. Small arteries (50–200 μm outer diameter) were analyzed in a blinded fashion and categorized as muscular, partly muscular, or non-muscular as previously described (Moral-Sanz et al., 2012; Morales-Cano et al., 2014). The medial wall thickness was calculated as external elastic lamina diameter minus the internal lamina diameter using ImageJ (Vs. 1.41, NIH, Bethesda, MD, United States of America).

#### Vascular reactivity

Intrapulmonary arteries were carefully dissected free of surrounding tissue from the inferior right lung lobe and cut into rings (1.8–2 mm length). PA rings were mounted on a wire myograph in Krebs physiological solution continuously bubbled with 21% O_2_, 5% CO_2_, and 74% N_2_ (Normoxia) and stretched to a transmural pressure equivalent to 30 mmHg, as previously described (Cogolludo et al., 2009; Morales-Cano et al., 2014). To confirm smooth muscle viability, arteries were first stimulated by raising the potassium concentration of the buffer to 80 mM and then allowed to recover. After washout, cumulative concentration responses curves to serotonin (5-HT, 30 nM–30 µM) were performed. Thereafter, concentration-response curves to acetylcholine (ACh; 1 nM−10 µM) and sodium nitroprusside (SNP, 10 pM–10 µM) were performed by cumulative addition to assess the endothelium-dependent and endothelium-independent vasodilatation, respectively.

In another set of experiments, rat PA rings derived from healthy animals were submaximally precontracted with the thromboxane A_2_ mimetic U46619 (0.1 µM), and cumulative concentration-response curves (10 nM–10 µM) to 5Z-7-oxozeaenol or riociguat in the absence or presence of 1 µM 5Z-7-oxozeaenol, were determined.

### Western blotting analysis

Lung homogenates were applied to a sulfate-polyacrylamide gel, and proteins were transferred to polyvinylidene difluoride membranes and incubated with primary antibodies directed against the following proteins: TAK-1, ERK1/2, p38 and JNK or the phosphorylated forms of ERK1/2, and JNK (all: Cell Signaling Technology, MA), p-TAK-1 (Abcam, United Kingdom), p-p38 (Santa Cruz, United States of America) or *β*-actin (Sigma Aldrich, Spain). Membranes were then incubated with secondary peroxidase-conjugated antibodies. Antibody binding was detected by an ECL system (Amersham Pharmacia Biotech, Amersham, United Kingdom), and images were acquired using Odyssey Fc System (Li-COR Biosciences, United States) and densitometry analysis performed using Quantity One software. Results were normalized by the relative expression of smooth muscle *β*-actin.

### *Analysis of IL-1*β *production*

Levels of IL-1β in whole lung homogenates were quantified by specific Rat DuoSet ELISA Development Systems (R&D System, United States) (Pandolfi et al., 2017).

### *Cell proliferation* and metabolic extraction

Rat PA smooth muscle cells (PASMCs) primary cell cultures were generated as previously described from SuHyp rats (Pandolfi et al., 2017). PASMC, seeded at 30.000 cell/ml (approximately 50% confluence) in 96-well plates, were growth arrested for 24 h in 0.1% fetal calf serum (FCS) DMEM medium and then exposed to 5Z-7-oxozeaenol, riociguat (1nM–1 µM) or vehicle for 48 h in 10% FCS or fresh 0.1% FCS. Cell proliferation was assessed using the BrdU incorporation assay. In another set of experiments, cells were washed with saline to remove residual medium and fixed with 10 ml each of ice-cold methanol, chloroform, and deionized water. Following phase separation, the aqueous phase was lyophilized and resuspended in 400 µl deuterium oxide (Cambridge Isotope Laboratories) for metabolic profiling.

### NMR data acquisition

Intact lung and RV tissue samples were examined by HR-MAS NMR using a Bruker AMX500 spectrometer (11.7 T). Samples were placed into a 50-μl zirconium oxide rotor using a rinsed cylindrical insert with 15 µl of a 0.1 mM solution of TSP in deuterium water (D2O) and spun at 4,000 Hz to remove the effects of spinning sidebands from the acquired spectra. Cell extracts were analyzed using a 500 MHz Bruker Ultrashield Plus spectrometer.

Shimming and NMR preparation times were reduced to a minimum, while the sample was chilled to 4°C to minimize metabolic changes. Several two-dimensional homonuclear and heteronuclear experiments, such as standard gradient-enhanced correlation spectroscopy (COSY), 1H–1H total correlated spectroscopy (TOCSY) and gradient-selected heteronuclear single quantum correlation (HSQC), were performed to carry out metabolomic assignments. A control 1H NMR spectrum was measured between consecutive two-dimensional (2D) spectra. No gross degradation was noted in the signals of multiple spectra acquired under the same conditions.

Standard solvent-suppressed spectra were grouped into 32,000 data points, averaged over 256 acquisitions. The data acquisition lasted a total of 15 min using a sequence based on the first increment of the nuclear Overhauser effect spectroscopy (NOESY) pulse sequence to suppress the effects of water. Sample acquisition was performed using a spectral width of 6,024 Hz prior to Fourier transformation, and the free induction decay (FID) signals were multiplied by an exponential weight function corresponding to a line broadening of 0.3 Hz. The spectra were referenced to the TSP singlet at a chemical shift of 0 ppm.

The NMR spectra were processed as described previously (Izquierdo-Garcia et al., 2011). Briefly, 1H-NMR spectra were binned to equal-length integral segments (*δ* = 0.01 ppm), and normalized to the total sum of the spectral regions.

#### Metabolomic analysis

Principal components analysis (PCA) and Partial Least Square Discriminant Analysis (PLS-DA) (Hotelling, 1933) were applied to 0.01 binned spectra to extract the most discriminative spectral subset from the total pool of metabolites and to remove outliers. The data obtained from this analysis were centered and scaled. The potential biomarkers selected from the PLS-DA loading matrix were confirmed by Hoteling’s T2 test (Hotelling, 1933). Multivariate statistical analysis was performed with ‘stats’ R package version 4.3.0 (Team, 2016). In addition, the resonances highlighted by the PCA were deconvoluted for metabolic quantification using the Global Spectral Deconvolution algorithm of MestRenova v. 8.1 (Mestrelab Research S.L., Santiago de Compostela, Spain) (Xia et al., 2009; Chong et al., 2018). The resonances were identified according to Chenomx NMR suit metabolic database (software version 9.0; Chenomx Inc. Edmonton, Canada)), the Human Metabolome Database (Wishart et al., 2012), and characteristic cross-peaks from 2D spectra to help in unequivocal assignation of these metabolites. Supplementary Table S1 display the complete list of metabolites assigned to detected NMR signals. For metabolic quantification, statistical significance was determined using a Bonferroni-corrected Student’s t-test (Vinaixa et al., 2012) assuming unequal variance, and variations were considered statistically significant when the adjusted *p*-value was less than 0.05.

### Metabolic pathways analyses

Metabolic pathway analysis was performed using the Pathway Analysis module (Xia and MetPA, 2010) of Metaboanalyst v.5.0 (Chong et al., 2018), which combines results from robust pathway enrichment analysis (Kankainen et al., 2011) with pathway topology analysis (Aittokallio and Schwikowski, 2006) to help researchers identify the most relevant pathways involved in the conditions under study. Briefly, pathway enrichment analysis examines whether metabolites in predefined pathways are at the top or bottom of a ranked list. In contrast, pathway topology analysis applies graph theory to measure the importance of an experimentally identified metabolite in a predefined metabolic pathway. KEGG metabolic pathways were used as the backend knowledgebase. The selected pathway enrichment analysis method was GlobalAncova (Hummel et al., 2008), node importance measure for topological analysis was out-degree centrality. Centrality is a standard metric used in graph theory to estimate the relative importance of individual nodes to the overall network (Liu et al., 2019). Out-degree is the number of outgoing links or successor nodes (Xia et al., 2009; Chong et al., 2018).

### Statistical analysis

Results are expressed as mean ± SEM. Technical replicates were averaged to provide a single data point before further analysis. Statistical analysis was performed using GraphPad Prism 5 as detailed in each figure legend. Individual cumulative concentration-response curves were fitted to a logistic equation and maximal responses (E_max_) and the negative log of IC_50_ (concentration of the drug that induced half-maximal response) were calculated. For multiple comparisons, normally distributed data were analyzed by one-way ANOVA followed by Dunnet’s *post hoc* test (Dunnett, 1955). For metabolic quantification, statistical significance was determined using a Bonferroni-corrected Student’s t-test (Vinaixa et al., 2012), assuming unequal variance. Adjusted *p*-value less than 0.05 was considered statistically significant.

## Results

*In vitro* characterization of the vasodilator and antiproliferative effects of riociguat and 5Z-7-oxozeaenol in pulmonary vascular tissues.

**FIGURE 1:**
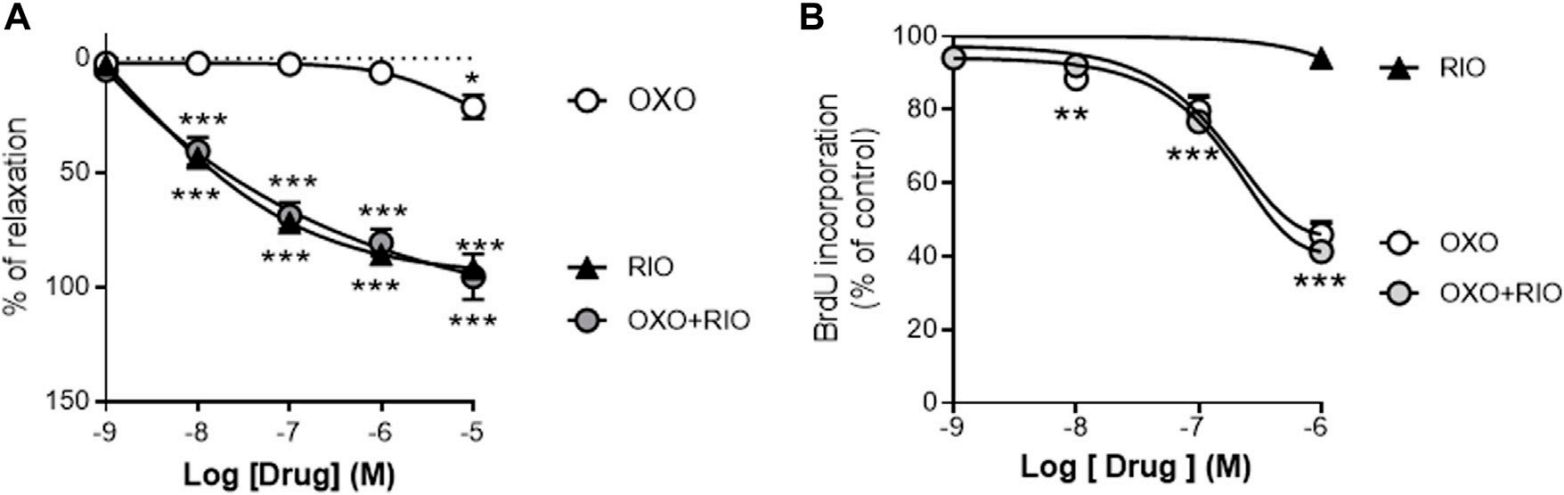
Vasodilator and antiproliferative effects of the TAK-1 inhibitor 5Z-7-oxozeaenol and the soluble guanylate cyclase activator riociguat in healthy rat pulmonary arteries. **(A)** Concentration-dependent relaxant effects of 5Z-7-oxozeaenol (OXO) and riociguat (RIO) in the absence or presence of OXO (1 µM) in PA stimulated with 0.1 µM of U46619. **(B)** Concentration-dependent effects of RIO and OXO in the absence or presence of RIO (1 µM) in the proliferation induced by 10% of FCS in PASMCs. *n* = 6-7 vascular rings **(A)** or experiments run in triplicate **(B)** for each group *p < 0,05 *versus* control (two-way ANOVA followed by Dunnett’s *post hoc* test).

We first analyzed the vasodilator and antiproliferative effects *in vitro* of the two drugs and the potential interactions between them. Riociguat induced a concentration-dependent relaxation in U46619-stimulated rat PA (Figure 1A; E_max_ = 92 ± 8%; pIC_50_ = 6.8 ± 0.4) but had negligible effects on the proliferation of rat PASMCs induced by 10% of FCS (Figure 1B). In contrast, the TAK-1 inhibitor 5Z-7-oxozeaenol only exerted a minor relaxant response at the highest concentration tested (10 μM; E_max_ = 21 ± 5%; Figure 1A) whereas it inhibited PASMCs proliferation in a concentration-dependent manner (E_max_ = 46 ± 3%; pIC_30_ = 6.9 ± 0.2; Figure 1B). Notably, neither 5Z-7-oxozeaenol (1 µM) affected the relaxant responses induced by riociguat (Figure 1A), nor activation of sGC modulated the antiproliferative effects exerted by 5Z-7-oxozeaenol (Figure 1B).

These *in vitro* studies suggested that riociguat acts mainly as a vasodilator agent whereas 5Z-7-oxozeaenol is an effective antiproliferative agent, targeting two central pathological features in PH, but there were no interactions between them. Therefore, we next investigated the potential therapeutic effects of 5Z-7-oxozeaenol as a single or as an add-on therapy to riociguat in the SuHyp rat *in vivo* model of PAH.

### *In vivo* study of riociguat and 5Z-7-oxozeaenol in the SuHyp rat model of PH

PH was induced in rats by a single injection of SU5416 (20 mg/kg, sc) and chronic exposure to normobaric hypoxia (10% O_2_) for 3 weeks (Supplementary Figure S1A). 2 weeks after SU5416 injection, rats started receiving daily intraperitoneal injections of vehicle, 5Z-7-oxozeaenol, riociguat or combined therapy for 1 week. Body weight in the SuHyp group was significantly smaller than in the normoxic control group (Supplementary Figure S1B). Treatment with 5Z-7-oxozeaenol, riociguat or combined therapy did not affect body weight compared to vehicle-treated SuHyp rats.

### Effects of riociguat and 5Z-7-oxozeaenol on pulmonary and cardiac hemodynamics

**FIGURE 2:**
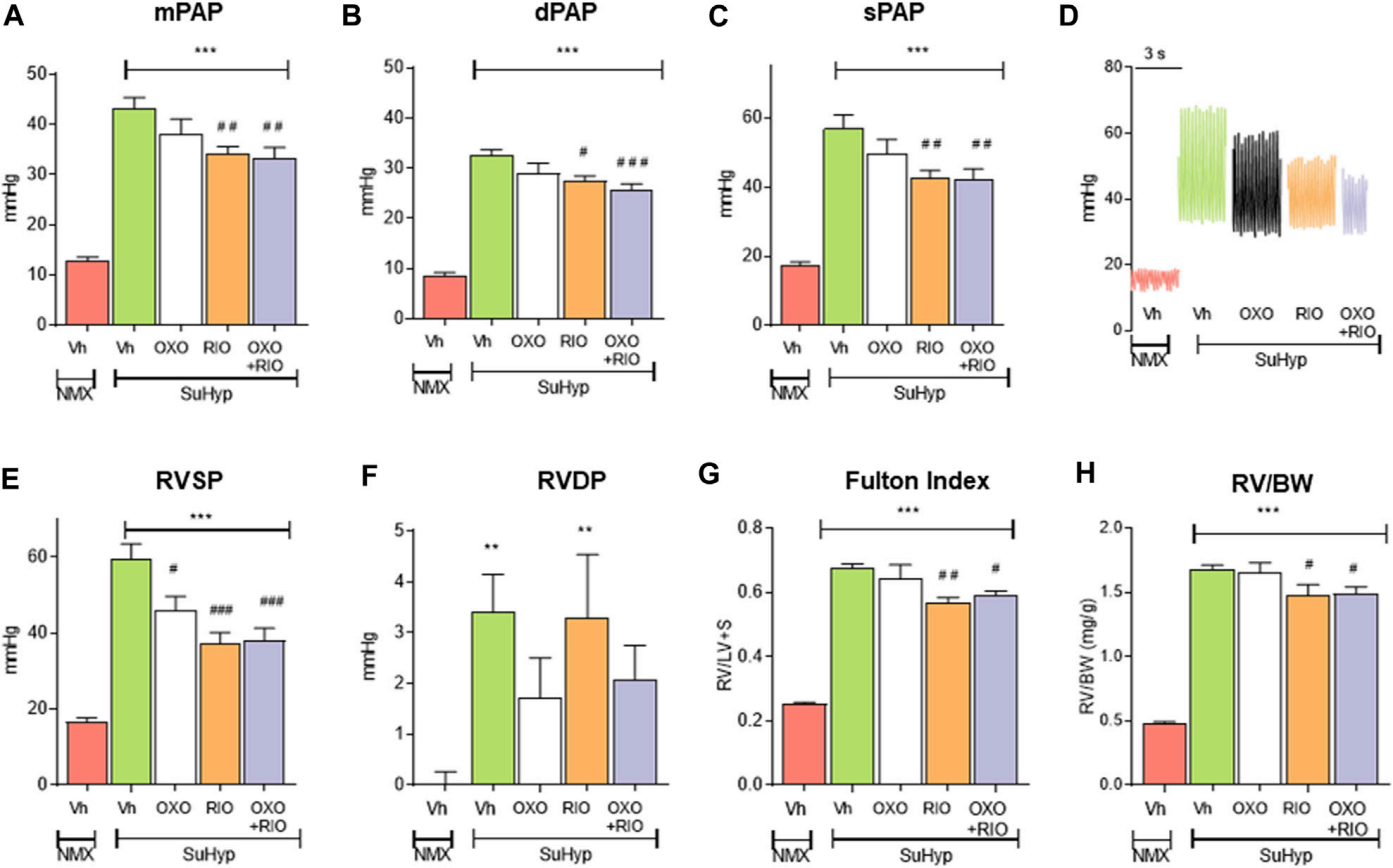
Treatment with riociguat alone or combined with 5-z-7oxozeaenol attenuates pulmonary hypertension in the Sugen 5426/hypoxia (SuHyp) rat model. **(A)** Mean pulmonary arterial pressure (mPAP), **(B)** diastolic PAP (dPAP) and **(C)** systolic PAP (sPAP) in control (NMX-Vh) and SuHyp rats treated with vehicle, 5Z-7-oxozeaenol (SuHyp-OXO; 3 mg·kg^–1^·day^–1^), riociguat (SuHyp-RIO; 3 mg·kg^–1^·day^–1^) or both drugs combined (SuHyp-OXO-RIO). **(D)** Representative PAP recordings in each treatment group. **(E)** Right systolic and **(F)** end-diastolic ventricular pressure (RVSP and RVEDP). **(G)** Fulton index (ratio between RV and left ventricle plus septum weight) and **(H)** right ventricular weight relative to total body weight in the different treatment groups. Each bar shows the mean +SEM (*n* = 6–10 animals in each group). *p < 0.05 versus NMX-Vh and ^#^ *p* < 0,05 *versus* SuHyp vehicle (One-way ANOVA followed by Bonferroni’s *post hoc* test).

Three weeks following SU5416 administration and chronic exposure to hypoxia, SuHyp rats developed PH with PAP mean values of 43 ± 2 mmHg (Figure 2). Some animals were sacrificed 2 weeks after administration of SU5416 to confirm the onset of development of PH prior to initiating interventional treatments (Mean PAP values 12 ± 0.7 mmHg vs. 23 ± 0.7 in the normoxic and SuHyp groups; *n* = 3; *p* < 0.005). Treatment with 5Z-7-oxozeaenol for 1 week had no effect on PAP (Figures 2A–D) but attenuated RV dysfunction, as evidenced by a significant reduction in RVSP and RVEDP (Figures 2E,F). In contrast, riociguat significantly reduced PAP and RVSP but did not modify RVEDP. Combined treatment with riociguat and 5Z-7-oxozeaenol resulted in a significant reduction in all these parameters but they remained above control values and no additive effects were observed. None of the treatments induced changes in heart rate (data not shown).

### Effects of riociguat and 5Z-7-oxozeaenol on cardiac hypertrophy and cardiac metabolic reprogramming

In line with the hemodynamic alterations described above, SuHyp rats developed a marked RV hypertrophy (Figures 2G,H). Riociguat induced a minor but significant reduction in the Fulton index, whereas treatment with 5Z-7-oxozeaenol neither induced effects on its own nor modified the effects induced by riociguat (Figures 2G,H).

**FIGURE 3:**
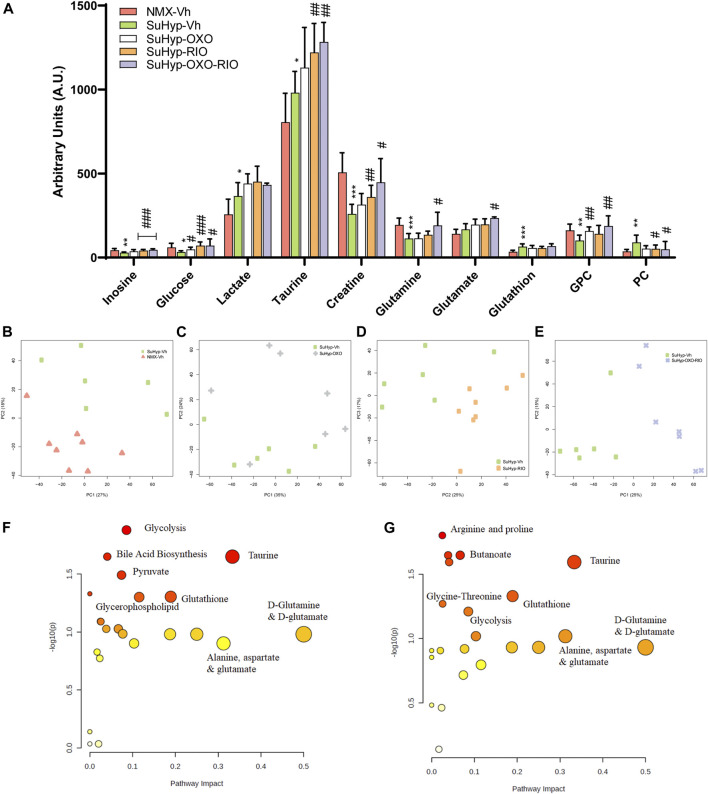
Treatment with riociguat and 5-Z-7oxozeaenol reduces metabolic reprogramming in the right ventricle (RV) from SuHyp rats. **(A)** Metabolomic profiling of RV samples showing the quantification of metabolites significantly altered between the five intervention groups. Statistical significance was determined using a Bonferroni-corrected Student’s t-test (*p < 0.05 *versus* normoxia + vehicle and #*p* < 0.05 *versus* SuHyp + vehicle). **(B)** Metabolic profiling of RV samples discriminated between SuHyp and control groups as shown by principal components analysis (PCA) performed on ^1^H-MRS data. Treatment with **(C)** 5Z-7-oxozeaenol, **(D)** riociguat or **(E)** both drugs combined induced a significant metabolic shift in the SuHyp RV. **(F)** Metabolic pathway analysis of the set of metabolites found to be significantly dysregulated between control and SuHyp rats **(G)** Incidence of the combined treatment with 5Z-7-oxozeaenol and riociguat on the SuHyp group over the specific metabolic pathways. *Y*-axis represents the statistical *p* values from enrichment analysis, and the *X*-axis represents the pathway impact value calculated from pathway topology analysis. The node colors represent the *p*-values (lowest: light yellow; highest: dark red) and the node radius indicate the pathway impact values. Dysregulated metabolic pathways are labeled. Control group: NMX-Vh, Sugen 5,416/hypoxia: SuHyp, SuHyp + 5Z-7-oxozeaenol treatment: SuHyp-OXO; SuHyp + riociguat treatment: SuHyp-RIO; SuHyp+ 5Z-7-oxozeaenol and riociguat treatment: SuHyp-OXO-RIO, PC: Principal Component, GPC: Glycero-phosphocholine and PC: Phosphocholine.

Metabolomic fingerprinting detected 10 metabolites significantly altered in the RV of SuHyp rats compared to control rats (Figure 3A and Supplementary Table S2). We found a full metabolic reprograming in SuHyp rats (Figure 3A) characterized by a shift in glucose metabolism, with higher metabolic concentrations of lactate and lower glucose; an increase of glutaminolysis with higher concentrations of glutamate and glutathione and lower concentration of glutamine; alterations in choline metabolism with a higher concentration of glycerophosphocholine (GPC) and lower of phosphocholine (PC). We also found an increase in taurine and GPC concentrations, whereas a decrease in inosine and creatine concentrations in SuHyp rats. PCA of RV NMR spectra provided perfect separation between SuHyp and control rats along the first two principal components (Figure 3B). The metabolites that changed between groups are highlighted in the PCA and PLS-DA loading plots shown in Supplementary Figures S2, 3. Metabolic pathway analysis was performed to identify the most relevant pathways involved in characteristic RV metabolic reprograming (Figure 3F). This pathway analysis identified alterations in energy metabolism, including glycolysis, Warburg effect and anaplerotic metabolism, amino acids biosynthesis and metabolism, glycerolipid metabolism and fatty acid degradation, and taurine metabolism.

Treatment with 5z-7-oxozeaenol, riociguat or both drugs combined, induced an apparent metabolic normalization in RV tissue (Figure 3A). Thus, PCA of RV NMR spectra provided clear discrimination between SuHyp and treated rats (Figures 3C–E). Treatment with 5Z-7-oxozeaenol induced the normalization of the metabolic levels of glucose, GPC, and PC whereas riociguat treatment induced a trend to normalize inosine, glucose, and GPC metabolic levels (Figure 3A). The combined treatment produced a further metabolic regularization characterized by the normalization of inosine, glucose, creatine, glutamine, GPC and PC metabolic levels. Finally, treatments with riociguat or with the combination of riociguat and 5Z-7-oxozeaenol induced an increment in taurine levels (Figure 3A). Metabolic pathway analysis highlighted that the combination of 5Z-7-oxozeaenol with riociguat induced a significant metabolic effect over taurine metabolism, amino acids biosynthesis and metabolism, glycolysis and TCA cycle metabolism *via* glycine, serine-threonine metabolism (Figure 3G).

### Effects of riociguat and 5Z-7-oxozeaenol on pulmonary vascular remodeling and lung metabolic reprogramming

**FIGURE 4:**
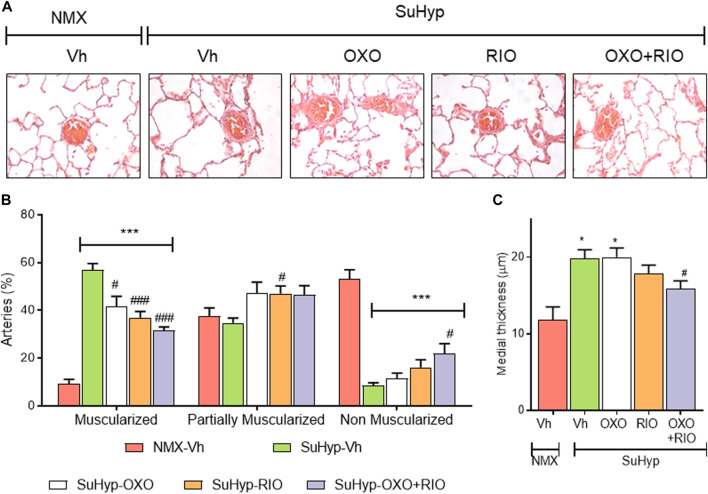
Riociguat and 5-z-7oxozeaenol exert additive effects on preventing pulmonary vascular remodeling. **(A)** Percentage of muscular, partially muscular and non-muscular arteries in the different treatment groups. **(B)** Medial wall thickness of pulmonary arteries (50–200 μm external diameter). **(C)** Representative images of lung histology. Each bar shows the mean +SEM (*n* = 5-6 animals in each group). *p < 0.05 *versus* normoxia + vehicle and #*p* < 0.05 *versus* hypoxia + vehicle (One-way ANOVA followed by Bonferroni’s *post hoc* test).

Lung histological evaluation confirmed the development of pulmonary vascular remodeling in SuHyp rats, with an increase in the percentage of muscular PA and the corresponding decrease in partially and non-muscular PA (Figures 4A,B) and a significant increase in the medial wall thickness of distal PA (Figure 4C). In contrast to our hemodynamic findings, combined therapy had an additive protective effect on pulmonary vascular remodeling. Thus, treatment with riociguat or 5Z-7-oxozeaenol significantly reduced the percentage of muscular PA but only combined treatment with riociguat plus 5Z-7-oxozeaenol significantly increased the percentage of non-muscular PA and reduced the medial wall thickness in SuHyp rats (Figure 4).

**FIGURE 5:**
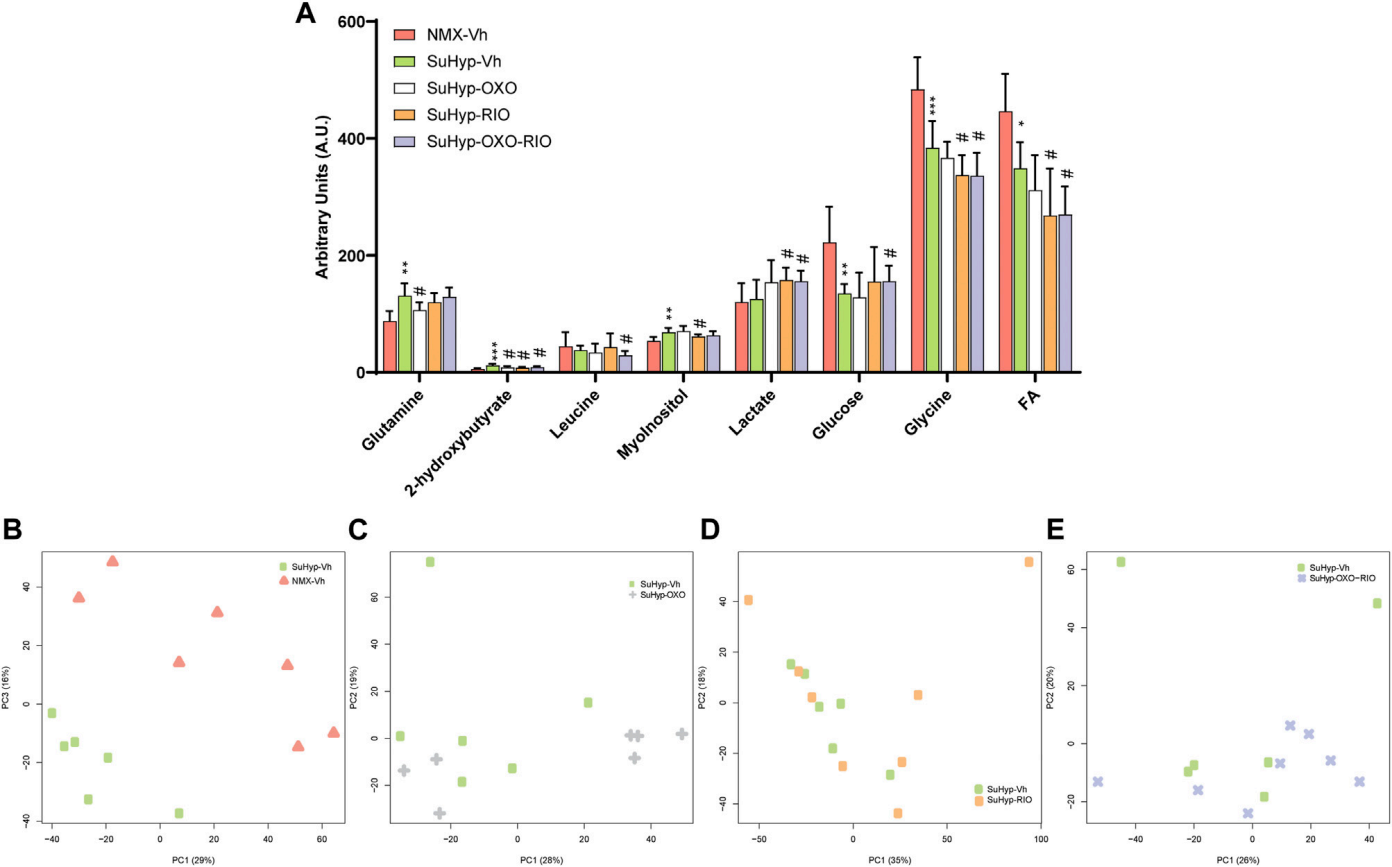
Treatment with riociguat and 5-z-7oxozeaenol has a modest effect on the lung metabolic reprogramming in the SuHyp rats. **(A)** Metabolomic profiling of lung samples showing the quantification of metabolites significantly altered between the five intervention groups. Statistical significance was determined using a Bonferroni-corrected Student’s t-test (*p < 0.05 *versus* normoxia vehicle and #*p* < 0.05 *versus* SuHyp vehicle). **(B)** Metabolic profiling of lung samples discriminated between SuHyp and control groups as shown by principal components analysis (PCA) performed on ^1^H-MRS data. Treatment with **(C)** 5Z-7-oxozeaenol, **(D)** riociguat or **(E)** both drugs induced a modest effect on the metabolic changes observed in the lungs from SuHPX rats. Control group: NMX-Vh, Sugen 5,416/hypoxia: SuHyp, SuHyp + 5Z-7-oxozeaenol treatment: SuHyp-OXO; SuHyp + riociguat treatment: SuHyp-RIO; SuHyp + 5Z-7-oxozeaenol and riociguat treatment: SuHyp-OXO-RIO, PC: Principal Component, FA: Fatty Acids.

Metabolomic fingerprinting of lung samples detected eight metabolites significantly altered (Figure 5A and Supplementary Table S2), enabling the discrimination between SuHyp and control groups (Figure 5B). The PCA and PLS-DA loading plots highlighted the most significant metabolites that distinguish between groups (Supplementary Figures S4, 5). Specifically, we observed a reduction in the concentration of glucose, glycine and free fatty acids, and an increase in alanine, glutamate, glutamine, creatine, threonine, myo-inositol, taurine, carnitine, valine, 2-hydroxybutyrate and leucine (Figure 5A). However, the metabolic impact of the intervention protocols in the lung (Figures 5C–E) was less than that detected in the RV tissue (Figure 3). 5Z-7-oxozeaenol treatment induced a trend to the normalization of glutamine levels and 2-hydroxybutyrate, whereas riociguat treatment induced the normalization of myo-inositol and 2-hydroxybutyrate. 5Z-7-oxozeaenol and riociguat combined treatment induced a significant trend to the normalization of glucose and 2-hydroxybutyrate concentrations. In addition, a significant decrease in glycine and free fatty acids, and an increase in lactate were detected after the treatment with riociguat, either alone or in combination with 5Z-7-oxozeaenol (Figure 5A).

### Effects of riociguat and 5Z-7-oxozeaenol on pulmonary vascular dysfunction

**FIGURE 6:**
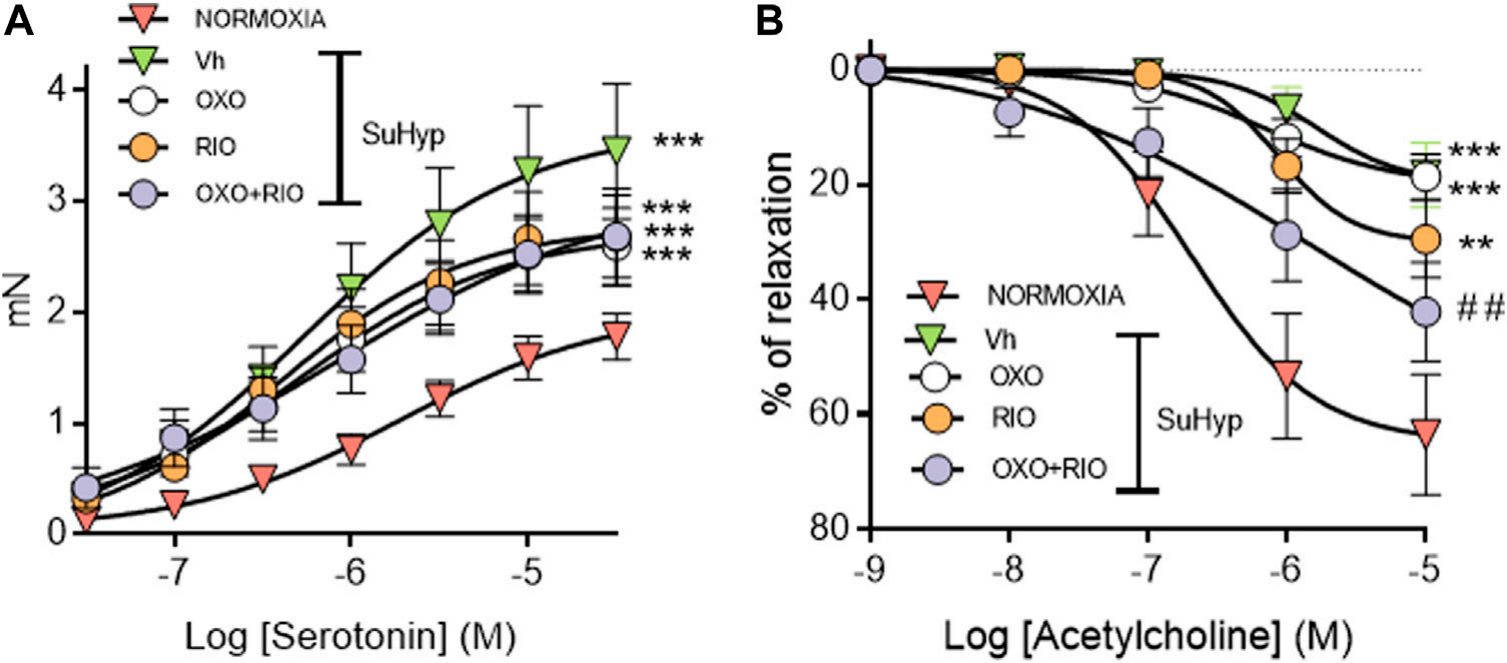
Riociguat plus 5-z-7oxozeaenol combination attenuates pulmonary endothelial dysfunction in rats with PH. **(A)** Contractile responses induced by serotonin is isolated intrapulmonary arteries from control (normoxic) rats and rats with PAH (SuHyp) treated with vehicle (Vh), 5-z-7-oxozeaenol (OXO), riociguat (RIO) or a combination of both drugs (OXO + RIO). **(B)** Concentration-dependent relaxant responses induced by the endothelium-dependent vasodilator acetylcholine in pulmonary arteries isolated from the different experimental groups. N = 12-15, *p < 0.05 *versus* normoxia and # vs. SuHyp + vehicle (two-way ANOVA followed by Bonferroni’s *post hoc* test).

PA from SuHyp rats exhibited a significant increase in the contractile responses induced by serotonin and a marked reduction in the relaxant responses induced by acetylcholine (Figure 6). Administration of riociguat, 5Z-7-oxozeaenol or the combination of both drugs did not reduce the hyperresponsiveness to serotonin (Figure 6A). In contrast, the combination of riociguat and 5Z-7-oxozeaenol attenuated the endothelial dysfunction induced by SuHyp, as evidenced by a significant increase in the relaxation induced by acetylcholine (Figure 6B).

### Effects of riociguat and 5Z-7-oxozeaenol on molecular markers of pulmonary hypertension

**FIGURE 7:**
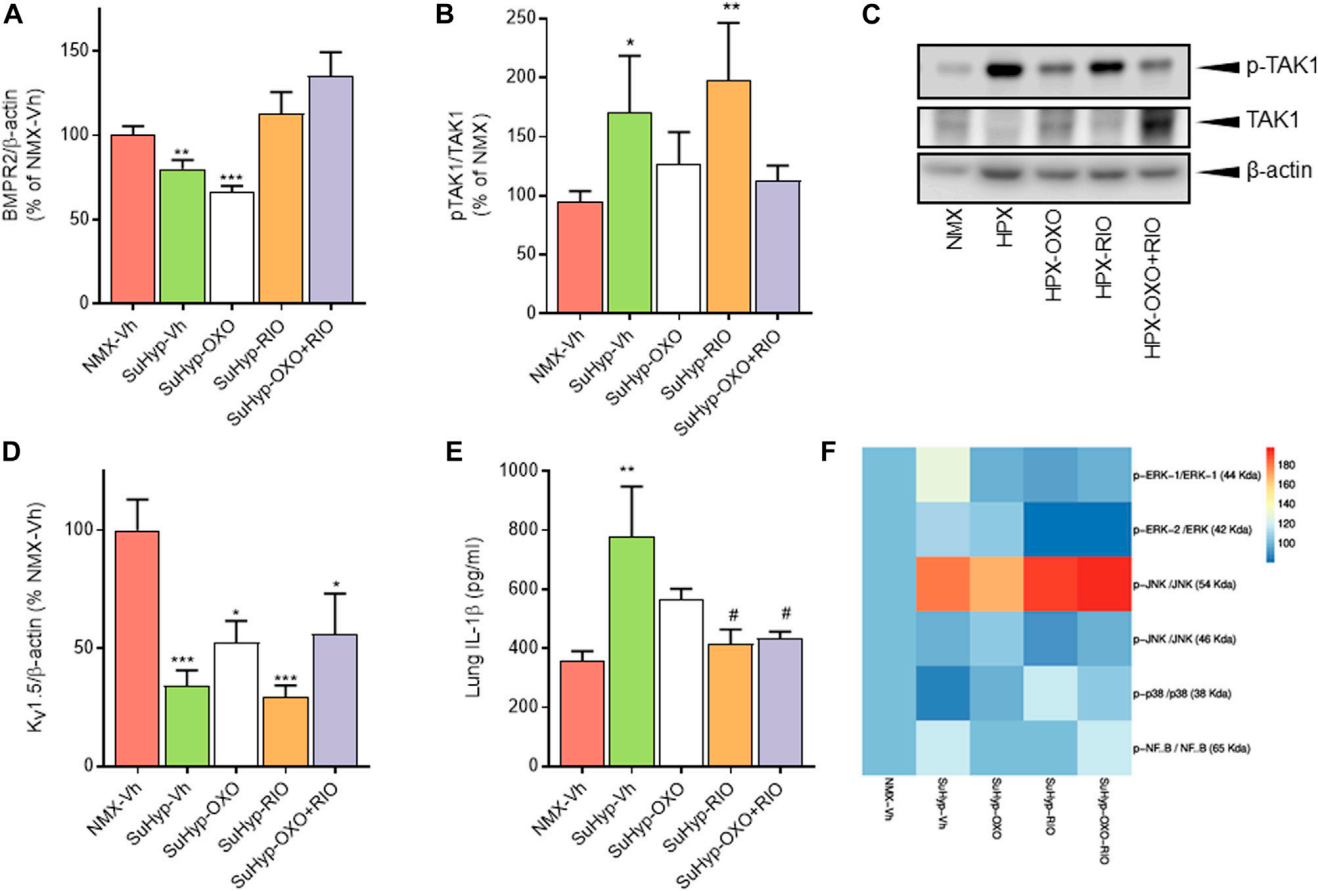
Treatment with riociguat and 5-z-7oxozeaenol restores the expression of BMPR2 and inhibits IL-β in the lungs from SuHyp rats. Densitometry analysis of **(A)** BMPR2/β-actin and **(B)** pTAK1/TAK1 protein expression in lung homogenates from control (normoxic) rats and rats with PAH (SuHyp) treated with vehicle (Vh), 5-z-7-oxozeaenol (OXO), riociguat (RIO) or a combination of both drugs (OXO + RIO). **(C)** Representative image of Western blot showing p-TAK1, TAK1 and *β*-actin. **(D)** Densitometry analysis of Kv1.5/β-actin in lung homogenates. **(E)** Quantification of IL-1β levels in lung homogenates in the five experimental groups. **(F)** Heatmap showing the densitometry analysis of Western blot performed to assess the activation of the NF-κB and MAPK signaling pathway. N = 6-10 animals in each group, *p < 0.05 *versus* normoxia and # vs. SuHyp + vehicle (One sample *t*-test).

To gain insight into the underlying pathways mediating the changes induced by our intervention protocol, we assessed the expression of molecular markers involved in the pathophysiology of PH, including those associated with the TGFβ signaling pathway, inflammation, or ionic remodeling (Rabinovitch, 2012; Humbert et al., 2019). As previously reported in the monocrotaline and chronic hypoxia PH rat models (Nasim et al., 2012), lungs from SuHyp rats also displayed reduced protein levels of BMPR2 and increased activated TAK-1 (Figures 7A,B). As expected, treatment with 5Z-7-oxozeaenol but not riociguat significantly reduced the levels of activated TAK1 (Figures 7B,C). In contrast, administration of riociguat but not 5Z-7-oxozeaenol resulted in an increase in lung BMPR2 levels, reaching values above those found in control animals (Figure 7A). Downregulation of voltage-gated K^+^ channels (K_V_) channels (notably K_V_ 1.5), is also considered to be an early contributor to the pathophysiology of PAH leading to vasoconstriction of PA, hypertrophy and remodeling (Antigny et al., 2016; Thenappan et al., 2018). In line with previous findings (Wang et al., 2005; Moral-Sanz et al., 2012; Morales-Cano et al., 2014; Mondejar-Parreno et al., 2019), K_V_ 1.5 protein expression was significantly reduced in the lungs from SuHyp rats (Figure 7D). However, none of the pharmacological treatments was able to restore the expression of these channels. Recently, the relevance of an immunoinflammatory component (characterized by infiltration and activation of macrophages and various T-lymphocyte subpopulations) in the development of PAH has also been demonstrated (Rabinovitch, 2012; Nicolls and Voelkel, 2017; Koudstaal et al., 2020). Thus, increased proinflammatory cytokines (IL-1β, IL-6, IFNγ or IL-17) have been associated with reduced quality of life and increased mortality (Chaouat et al., 2009; Pellicelli et al., 2010; Soon et al., 2010). Accordingly, lungs from SuHyp rats exhibited significantly increased levels of IL-1β (Figure 7E). Treatment with 5Z-7-oxozeaenol induced a modest reduction of IL-1β whereas treatment with riociguat, either alone or combined with the TAK1 inhibitor, fully abolished the increase in pulmonary IL-1β. Next, we examined the activation of two common inflammatory signaling pathways, nuclear factor-kappa B (NF-κB) and mitogen-activated protein kinases (MAPK) pathways (Shi and Sun, 2018). To evaluate the activation of MAPK and NF-*κ*B signaling pathways, we measured p38, extracellular signal-regulated kinases (ERK), c-Jun N-terminal kinases (JNK) and NF-*κ*B p65 protein phosphorylation by Western blot analysis. As shown in Figure 7F, only JNK was found to be activated in the lungs from SuHyp rats but treatment with 5Z-7-oxozeaenol or riociguat further increased the phosphorylation rate of this kinase. A rising trend in ERK1 and NF-*κ*B p65 subunit phosphorylation was also observed in the lungs from SuHyp rats but not in those treated with 5Z-7-oxozeaenol or riociguat.

### Effects of 5Z-7-oxozeaenol and riociguat on the metabolic reprogramming in PASMCs

**FIGURE 8:**
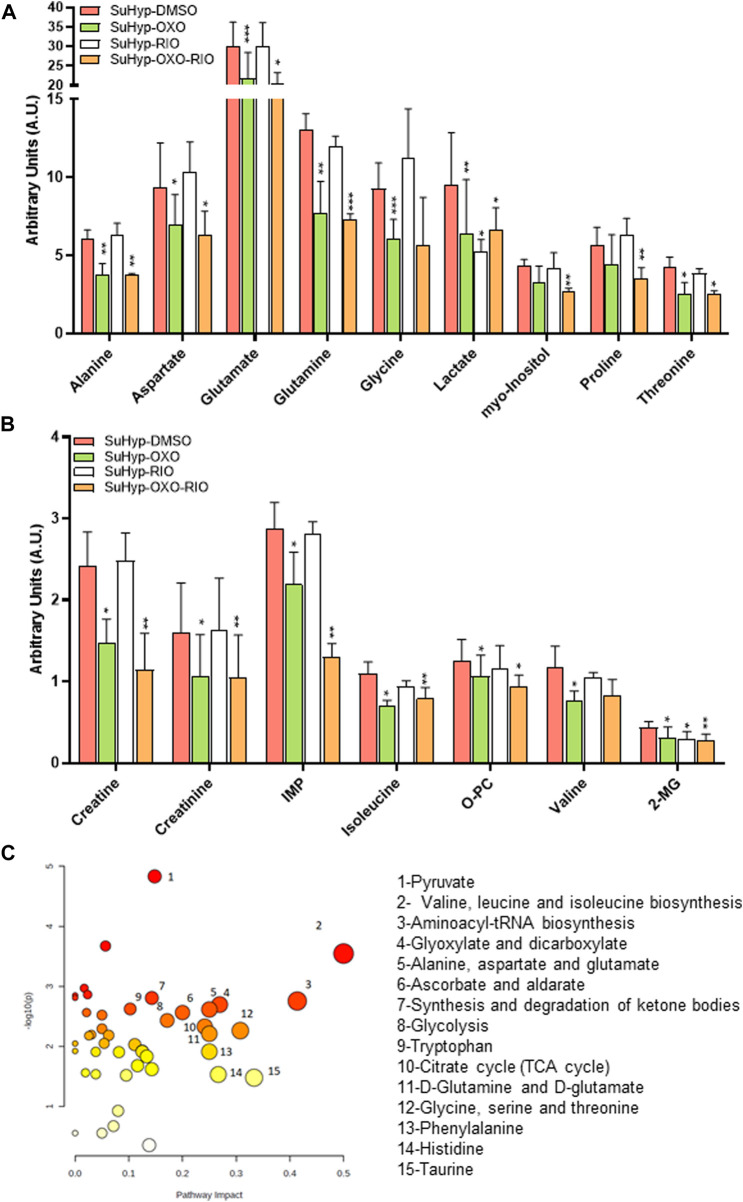
*Ex vivo* treatment with 5-z-7oxozeaenol attenuates the metabolic reprogramming in cultured pulmonary artery smooth muscle cells (PASMCs) from SuHyp rats. **(A,B)** Metabolomic profiling of cell extracts showing the quantification of metabolites significantly altered in PASMCs isolated from SuHyp rats and treated with vehicle (DMSO), 5Z-7-oxozeaenol (OXO; 1 µM), riociguat (RIO; 1 µM) or both drugs combined (OXO + RIO). Statistical significance was determined using a Bonferroni-corrected Student’s t-test (*p < 0.05 *versus* normoxia + vehicle and #*p* < 0.05 *versus* SuHyp + vehicle). **(C)** Metabolic pathway analysis of the set of metabolites found to be significantly dysregulated following treatment with the combination of OXO and RIO on PASMCs isolated from SuHyp rats. *Y*-axis represents the statistical *p* values from enrichment analysis, and the *X*-axis represents the pathway impact value calculated from pathway topology analysis. The node colors represent the *p*-values (lowest: light yellow; highest: dark red) and the node radius indicate the pathway impact values. Dysregulated metabolic pathways are labeled.

To further characterize the metabolic effects of 5Z-7-oxozeaenol and riociguat, primary cultures of PASMC were established from SuHyp rats and treated *in vitro* with both drugs. Treatment with 5Z-7-oxozeaenol, significantly attenuated the metabolic shift induced by SuHyp. Specifically, alanine, aspartate, creatine, creatinine, glutamate, glutamine, glycine, inosine monophosphate, 2-methylglutarate, isoleucine, lactate, PC, threonine and valine metabolic pools were found significantly decreased after the exposition to 5Z-7-oxozeaenol or the combination of 5Z-7-oxozeaenol and riociguat (Figures 8A,B). 5Z-7-oxozeaenol and riociguat combined treatment also induced a decrease in myo-inositol and proline concentrations. In contrast, treatment with riociguat only induced a decrease in lactate concentration (Figure 8A). Metabolic pathway analysis was performed to identify the most relevant pathways affected by 5Z-7-oxozeaenol and riociguat (Figure 8C). This pathway analysis identified alterations in energy metabolism including glycolysis and pyruvate metabolism, amino acids biosynthesis, TCA cycle metabolism *via* glycine-serine-threonine metabolism, and anaplerotic metabolism, including glutaminolysis, and ketone bodies metabolism.

## Discussion

Although several new therapies have become available over the last 2 decades, there is no cure for PAH, and the prognosis remains poor. In this study, we investigated the therapeutic efficacy of the combination of the TAK-1 inhibitor 5Z-7-oxozeaenol and the sGC stimulator riociguat in the experimental model of PH induced by the combined exposure to the VEGF receptor antagonist SU5416 and chronic hypoxia. We first demonstrated that *in vitro*, riociguat acts mainly as a vasodilator agent whereas 5Z-7-oxozeaenol is an effective antiproliferative drug. *In vivo,* treatment with 3 mg/kg riociguat for 1 week induced a modest reduction in PAP, pulmonary vascular remodeling and RV hypertrophy accompanied by an increase in BMPR2 expression and a reduction in lung inflammation. Riociguat also induced a partial reduction in metabolic reprogramming of RV and PASMCs. By contrast, the TAK-1 inhibitor 5Z-7-oxozeaenol significantly reduced pulmonary vascular remodeling and limited lung inflammation, induced a modest improvement in vascular remodeling and attenuated the metabolic reprogramming in RV and PASMCs in the rat SuHyp model of PAH. However, 5Z-7-oxozeaenol failed to reduce PAP or RV hypertrophy, did not increase BMPR2 expression and only had a modest metabolic effect in lung tissue. Importantly, combination therapy significantly improved hemodynamic parameters and cardiac hypertrophy, but no additive effects were observed. In contrast, the combination of riociguat and 5Z-7-oxozeaenol was associated with a greater reduction in pulmonary vascular remodeling and endothelial dysfunction than those observed with either drug alone. Moreover, the combined treatment was also associated with more pronounced metabolic effects, leading to the normalization of the metabolic dysregulation found in the RV and PASMCs of PH rats.

Riociguat isa sGC stimulator and is the only drug approved for both PAH and chronic thromboembolic pulmonary hypertension (CTEPH) (Ghofrani et al., 2013; Simonneau et al., 2015). sGC stimulators can directly stimulate the sGC enzyme in a NO‐independent fashion and produce their sensitization to low endogenous NO levels, increasing cGMP biosynthesis and leading to vascular relaxation and antiproliferative effects (Cogolludo et al., 2007). In the present study, we confirmed the ability of riociguat to induce almost full relaxation in isolated rat PA. These results are in line with our previous report showing the efficacy of riociguat as a vasodilator in isolated human and rat PA under various oxygen conditions (Morales-Cano et al., 2021). Surprisingly, riociguat had no significant effects in the proliferative ability of PASMCs from neither control nor SuHyp rats nor human PASMCs (unpublished observations). *In vitro* antiproliferative effects by other direct stimulators of sGC, such as YC-1 or BAY 41‐2,272, have been previously demonstrated (Huh et al., 2011; Joshi et al., 2011). However, studies assessing the *in vitro* antiproliferative capacity of riociguat are scarce. Thus, Patel *et al.*, reported the ability of riociguat to inhibit ET-1-induced growth of PASMCs derived from PAH patients (Patel et al., 2014). In contrast, other groups, including ours, did not find a significant attenuation of cell proliferation at concentration at which riociguat induces strong vasodilator effects (Morales-Cano et al., 2015; Nawa et al., 2016). In contrast, we found that low-dose of riociguat (3 mg/kg/day) reduced the increase in PAP, RV hypertrophy, and pulmonary vascular remodeling in the rat SuHyp model of PAH. These findings are consistent with those reported by other groups in different experimental models of PH (Schermuly et al., 2008; Lang et al., 2012; Rai et al., 2018) but suggest that the ability of riociguat to reduce the progression of pulmonary vascular remodeling and RV hypertrophy are strongly associated with its ability to reduce PAP rather than with direct antiproliferative effects.

5Z-7-oxozeaenol, a resorcylic acid lactone derived from fungus, is a potent and selective inhibitor of TAK1 (Ninomiya-Tsuji et al., 2003). TAK1, also known as MAP3K7, is a key member of the TGFβ non-canonical signaling pathway (Sato et al., 2005; Mihaly et al., 2014). A role for TAK1 in the pathogenesis of PAH has been proposed (Nasim et al., 2012; Dalvi et al., 2017). Thus, Nassim et al., demonstrated that BMPR-II dysfunction promotes the activation of TAK1 leading to aberrant activation of the TGFβ-MAPK axis in the lung and resulting in a pro-proliferative and anti-apoptotic response (Nasim et al., 2012). Notably, TAK1 expression and activity were shown to be increased in murine PASMCs carrying a PAH-associated *bmpr*2 non-sense mutation but also in hypoxia and monocrotaline-induced PAH rat models, which display reduced levels of *bmpr2* transcripts. Prof. Schneider’s group showed that an activating mutation of TAK1 is sufficient to induce myocardial hypertrophy and fulminant heart failure (Zhang et al., 2000). In the present study, we found that phosphorylated TAK1 is also increased in the lungs from SuHyp rats and that treatment with the TAK1 inhibitor 5Z-7-Oxozeanol significantly reduced PASMCs proliferation *in vitro* and limited pulmonary vascular remodeling *in vivo*. However, 5Z-7-Oxozeanol lacked acute vasodilator effects and it failed to reduce PAP or to limit RV hypertrophy in the SuHyp model. These results align with those found with sotatercept analogues (Yung et al., 2020; Joshi et al., 2022). Thus, despite neutralizing antibodies against activins induced modest effects on PAP, treatment with these analogues caused regression of vascular remodeling either as monotherapy or combined with a vasodilator.

Our results are also consistent with findings from other groups demonstrating the antiproliferative effects of TAK-1 inhibitors in several cell types (Nasim et al., 2012; Zippel et al., 2013; Song et al., 2014; Chen et al., 2016; Pandolfi et al., 2017) in the absence of significant hemodynamic effects (Garfield et al., 2019). Thus, Garfield et al. recently reported that 5Z-7-oxozeaenol had no effect on RVSP or Fulton index but prevented muscle loss in monocrotaline-treated rats (Garfield et al., 2019). Since the interventional treatments were initiated after the onset of pulmonary hypertension and were carried out only for 1 week, it remains to be determined whether treatment over a longer period of time would induce more robust hemodynamic effects. However, our study identifies novel protective effects induced by TAK-1 inhibition in the RV metabolic changes which could also contribute to alleviate PH-related RV dysfunction (Figures 2, 3).

Metabolic reprogramming in PAH is a major factor in the pathogenesis of the pulmonary vascular disease (Assad and Hemnes, 2015; Xu et al., 2021). The pulmonary vasculature in PAH shows normoxic activation of hypoxia-inducible factor 1-alpha (HIF-1α), which creates a ¨pseudo-hypoxic¨ environment despite normal oxygen supply T (Ryan and Archer, 2015). HIF-1α activation induces a metabolic shift toward aerobic glycolysis and a reduction of glucose flux into the mitochondria. To maintain mitochondria activity, metabolism through anaplerotic pathways increases, including fatty acid oxidation and choline-conjugated phospholipid metabolism (Sutendra et al., 2010; Kudryashova et al., 2015) and glutaminolysis (Egnatchik et al., 2017). In addition, RV hypertrophy induced by the sustained PH is characterized by a similar metabolic reprograming (Graham et al., 2018), although these changes are likely induced by ischemia rather than by impaired oxygen sensing (Ryan and Archer, 2015). Our findings in the rat SuHyp model reproduce the main metabolic alterations previously reported in lung and RV tissue in several PAH animal models (Zungu-Edmondson et al., 2017; Graham et al., 2018; Izquierdo-Garcia et al., 2018; Sakao et al., 2021). In the lung tissue, we confirmed: I) the enhancement of the glycolytic pathway, including lower glucose and higher lactate concentrations; II) alteration of one-carbon metabolism including higher glycine metabolism; III) upregulation of anaplerotic pathways, including glutaminolysis and upregulation of fatty acid oxidation. In RV tissue, we reproduced: I) the Warburg effect characterized by lower glucose and higher lactate concentrations; II) increase of anaplerotic metabolism including amino acids metabolism, glycerolipid metabolism and fatty acid degradation; III) and taurine metabolism.

Our data revealed that the impact of treatment with riociguat on lung tissue metabolism is limited inducing only a significant reduction in the concentration of myo-inositol, 2-hydroxybutyrate and leucine in the lungs from SuHyp rats. Myo-inositol has been proposed to modulate reduced interleukin-6 levels (Bizzarri et al., 2020), a cytokine involved in the development and progression of (Steiner et al., 2009), 2-hydroxybutyrate is a marker of the ketone metabolism that characterizes PAH (Mey et al., 2020), whereas leucine reduction may be related to glutamine metabolism (Bertero et al., 2019). The effect of riociguat in cultured rat PASMC is also scarce since we only found a reduction in lactate concentration but no other metabolites of the glycolytic pathway were normalized. In contrast, treatment with riociguat induced protective effects in RV metabolic disruption, inducing the normalization of choline-conjugated phospholipid metabolism, inosine, creatine, and glucose levels. Since heart failure is characterized by reduced myocardial inosine and creatine (Archer et al., 1995; Luptak et al., 2018), the normalization of these metabolic concentrations may be markers of an improvement of heart function following treatment with riociguat. To the best of our knowledge, this is the first report demonstrating a protective effect of riociguat on PAH metabolic alterations.

Treatment with 5Z-7-oxozeaenol in rat PASMC induced a significant effect in several metabolic pathways, including in glycolysis, citrate cycle, glutaminolysis, or branched-chain amino acids biosynthesis (Figure 8C), suggesting a protective effect on the metabolic dysregulation characteristic found in PH patients. For example, the reduction of lactate, and alanine concentrations is related to the normalization of the glycolysis pathway. Lower glutamine, glutamate and PC concentrations results from a lesser requirement of metabolic supplies by anaplerotic pathways. However, treatment with 5Z-7-oxozeaenol only normalized glutamine levels in lung tissue suggesting that its effects are more moderate *in vivo.* By contrast, 5Z-7-oxozeaenol induced the normalization of glucose and choline-conjugated phospholipid metabolism in RV tissue.

The combined treatment of 5Z-7-oxozeaenol and riociguat reinforces the metabolic effects of both treatments separately. In rat PASMC, combined treatment reproduces the metabolic alteration caused by 5Z-7-oxozeaenol treatment, but we also observed a statically significant reduction in glutamate, myo-inositol and proline concentrations. In lung tissue, combined treatment did not improve the metabolic effect of riociguat treatment alone. However, the combined treatment potentiates the metabolic effect induced by each drug alone in the RV tissue. Indeed, the levels of creatine and inosine from animals treated with 5Z-7-oxozeaenol and riociguat are more similar to the levels detected in normoxic animals than from animals treated with riociguat. These metabolic markers may reflect a better cardiac function in combined treatment animals.

In summary, the addition of the TAK1 inhibitor 5Z-7-oxozeaenol to riociguat significantly improved hemodynamic parameters and cardiac hypertrophy in the SuHyp rat model. Greater protective effects on pulmonary vascular remodeling and endothelial dysfunction were observed when both drugs were combined. Moreover, only the combination of riociguat with 5Z-7-oxozeaenol was able to normalize the metabolic dysregulation found in the RV rats with PAH suggesting that the inhibition of TAK1 might represent a cardioprotective target for the treatment of RV failure associated to PAH.

## Data Availability

The raw data supporting the conclusion of this article will be made available by the authors, without undue reservation.
