# Corrigendum: Impact of a TAK-1 inhibitor as a single or as an add-on therapy to riociguat on the metabolic reprograming and pulmonary hypertension in the SUGEN5416/hypoxia rat model

**DOI:** 10.3389/fphar.2023.1228923

**Published:** 2023-06-12

**Authors:** Daniel Morales-Cano, Jose Luis Izquierdo-García, Bianca Barreira, Sergio Esquivel-Ruiz, Maria Callejo, Rachele Pandolfi, Palmira Villa-Valverde, Ignacio Rodríguez, Angel Cogolludo, Jesus Ruiz-Cabello, Francisco Perez-Vizcaino, Laura Moreno

**Affiliations:** ^1^ Department of Pharmacology and Toxicology, School of Medicine, Universidad Complutense de Madrid, Madrid, Spain; ^2^ Ciber Enfermedades Respiratorias (Ciberes), Madrid, Spain; ^3^ Instituto de Investigación Sanitaria Gregorio Marañón, Madrid, Spain; ^4^ Centro Nacional de Investigaciones Cardiovasculares (CNIC), Madrid, Spain; ^5^ Department of Clinical Medicine, Aarhus University, Aarhus, Denmark; ^6^ Department of Chemistry in Pharmaceutical Sciences, School of Pharmacy, Universidad Complutense de Madrid, Madrid, Spain; ^7^ Instituto Pluridisciplinar, Universidad Complutense de Madrid, Madrid, Spain; ^8^ ICTS Bioimagen Complutense, Universidad Complutense de Madrid, Madrid, Spain; ^9^ Center for Cooperative Research in Biomaterials (CIC biomaGUNE), Basque Research and Technology Alliance (BRTA), Donostia San Sebastián, Spain

**Keywords:** pulmonary hypertension, antiproliferative, metabolomics, combination therapy, right ventricle

In the published article, there was an error in Figure 2 as published. There was a mistake in the identification of two of the panels (B and C) which were described respectively as “(B) systolic PAP (sPAP) and (C) diastolic PAP (dPAP).” The corrected Figure 2, and it is caption, appear below.

**FIGURE 2:**
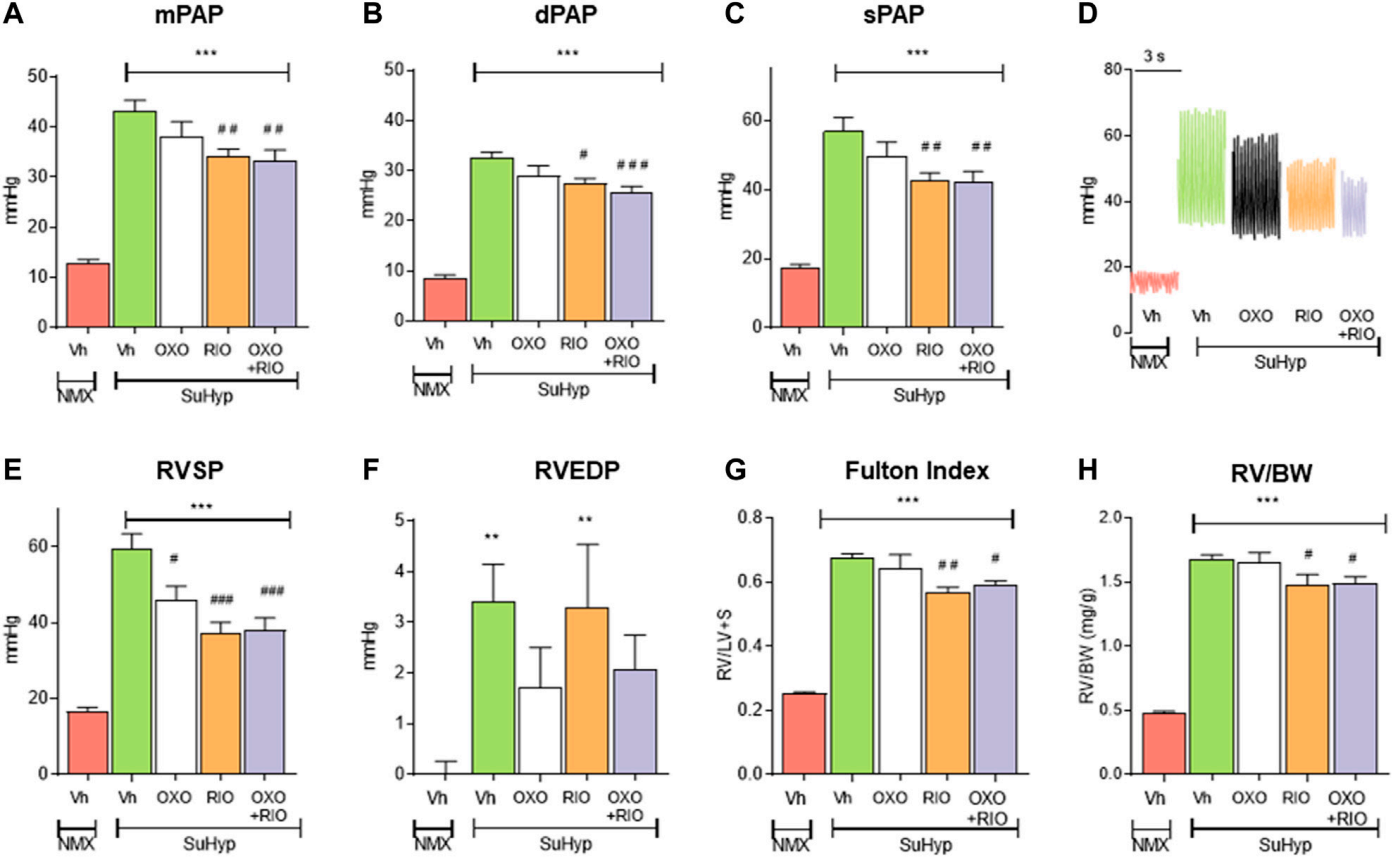
Treatment with riociguat alone or combined with 5-z-7oxozeaenol attenuates pulmonary hypertension in the Sugen 5426/hypoxia (SuHyp) rat model. **(A)** Mean pulmonary arterial pressure (mPAP), **(B)** diastolic PAP (dPAP) and **(C)** systolic PAP (sPAP) in control (NMX-Vh) and SuHyp rats treated with vehicle, 5Z-7-oxozeaenol (SuHyp-OXO; 3 mg·kg^−1^·day^−1^), riociguat (SuHyp-RIO; 3 mg·kg^−1^·day^−1^) or both drugs combined (SuHyp-OXO-RIO). **(D)** Representative PAP recordings in each treatment group. **(E)** Right systolic and **(F)** end-diastolic ventricular pressure (RVSP and RVEDP). **(G)** Fulton index (ratio between RV and left ventricle plus septum weight) and **(H)** right ventricular weight relative to total body weight in the different treatment groups. Each bar shows the mean +SEM (*n* = 6–10 animals in each group). *p < 0.05 *versus* NMX-Vh and #*p* < 0,05 *versus* SuHyp vehicle (One-way ANOVA followed by Bonferroni´s *post hoc* test).

The authors apologize for this error and state that this does not change the scientific conclusions of the article in any way. The original article has been updated.

