# Supplementary material for: Impact of a TAK-1 inhibitor as a single or as an add-on therapy to riociguat on the metabolic reprograming and pulmonary hypertension in the SUGEN5416/hypoxia rat model
Source: Front Pharmacol. 2023 Mar 29;14:1021535. doi: 10.3389/fphar.2023.1021535 (PMC10090662; doi:10.3389/fphar.2023.1021535)

Supplementary Material

Impact of a TAK-1 inhibitor as a single or as an add-on therapy to riociguat on the metabolic reprograming and pulmonary hypertension in the SUGEN5416/hypoxia rat model.

Daniel Morales-Cano[1, 2, 3, 4, 5] †, Jose Luis Izquierdo-García [2, 6,7] †, Bianca Barreira [1, 2, 3], Sergio Esquivel-Ruiz [1, 2, 3], Maria Callejo [1, 2, 3], Rachele Pandolfi[1, 2, 3], Palmira Villa-Valverde[2,8], Ignacio Rodríguez[2,6], Angel Cogolludo[1, 2, 3], Jesus Ruiz-Cabello[2, 6,9 #], Francisco Perez-Vizcaino[1, 2, 3 #], Laura Moreno[1, 2, 3 #*].

Table S1: Metabolites identified in NMR spectrum from Right Ventricle and Lung tissue samples.

Metabolite	NMR signal (ppm)	Right Ventricle Tissue	Lung Tissue
2-Aminobutyrate	3.7, 1.9, 1.0	Assigned	Assigned
2-Hydroxybutyrate	4.0, 1.7, 1.6, 0.9	No Assigned	Assigned
3-Aminoisobutyrate	3.1, 3.0, 2.6, 1.2	Assigned	Assigned
3-Hydroxybutyrate	4.1, 2.4, 2.3, 1.2	Assigned	Assigned
Acetate	1.9	Assigned	Assigned
Acetone	2.2	Assigned	Assigned
Alanine	3.8, 1.5	Assigned	Assigned
Carnitine	4.6, 3.4, 3.2, 2.4	No Assigned	Assigned
Choline	4.1, 3.5, 3.2	Assigned	Assigned
Creatine	3.9, 3.0	Assigned	Assigned
Dimethyl sulfone	3.2	Assigned	Assigned
Fumarate	6.5	Assigned	Assigned
Glucose	4.6, 3.8, 3.5, 3.2	Assigned	Assigned
Glutamate	3.8, 2.4, 2.1	Assigned	Assigned
Glutamine	7.6, 6.9, 3.8, 2.4, 2.2,	Assigned	Assigned
Glutathione	8.5, 8.2, 4.6, 3.8, 3.0, 2.5, 2.2	Assigned	Assigned
Glycerol	3.8, 3.7, 3.5	Assigned	Assigned
Glycine	3.6	Assigned	Assigned
Inosine	8.4, 8.2, 4.8, 4.4, 4.3, 3.9, 3.8	Assigned	Assigned
Isoleucine	3.7, 2.0, 1.5, 1.2, 1.0	Assigned	Assigned
Lactate	4.1, 1.3	Assigned	Assigned
Leucine	3.7, 1.7, 1.0	No Assigned	Assigned
Nicotinurate	8.9, 8.8, 8.7, 8.2, 7.6, 4.0	Assigned	Assigned
O-Phosphocholine	4.2, 3.6, 3.2	Assigned	Assigned
Sarcosine	3.6, 2.7	Assigned	Assigned
sn-Glycero-3-phosphocholine	4.3, 3.9, 3.7, 3.6, 3.2	Assigned	Assigned
Succinate	2.4	No Assigned	Assigned
Taurine	3.4, 3.3	Assigned	Assigned
Threonine	4.3, 3.6, 1.3	No Assigned	Assigned
Valine	3.6, 2.3, 1.0	Assigned	Assigned

Table S2: | Summary of postulated metabolites and their relative amounts from normoxia and SuHyp groups.

	Metabolite	Normoxia (A.U.)		SuHyp (A.U.)		Bonferroni corrected p-val
		Mean	SD	Mean	SD	
**Right Ventricle tissue**	Acetate	28,30	6,05	27,87	6,77	9,47E-01
	Alanine	114,22	13,22	109,71	13,51	7,36E-01
	Choline	15,35	4,04	14,15	2,42	8,15E-01
	Creatine	507,35	58,07	259,23	28,49	1,09E-04
	Glucose	60,17	12,39	33,11	3,39	1,37E-02
	Glutamate	140,65	13,83	167,28	16,88	9,42E-02
	Glutamine	193,85	20,49	113,44	14,60	4,74E-04
	Glutathion	34,24	4,53	65,01	8,02	1,41E-04
	sn-Glycero-3-phosphocholine	161,21	18,82	100,94	16,11	3,66E-03
	Inosine	43,54	5,07	28,90	2,21	2,82E-03
	Lactate	257,02	45,13	365,50	40,05	2,22E-02
	O-Phosphocholine	36,36	5,75	89,77	21,60	1,85E-03
	Taurine	805,34	86,20	980,91	63,60	3,73E-02
**Lung Tissue**	2-hydroxybutyrate	5,87	0,60	11,66	1,38	8,27E-04
	Alanine	134,61	10,49	221,77	12,08	5,55E-05
	Carnitine	212,88	31,60	279,83	15,79	4,27E-02
	Creatine	71,74	5,46	96,64	3,95	1,10E-03
	FA	446,43	31,96	348,86	22,27	1,19E-02
	Glucose	134,06	17,68	76,53	5,69	3,53E-03
	Glutamate	197,18	9,56	267,48	8,06	4,27E-05
	Glutamine	87,69	8,66	131,12	10,52	2,95E-03
	Glycine	364,87	22,63	279,16	26,66	1,33E-02
	Leucine	37,90	4,59	58,55	7,49	1,65E-02
	MyoInositol	53,60	3,47	68,36	3,88	6,04E-03
	Taurine	131,89	10,28	161,14	8,72	2,40E-02
	Threonine	55,21	6,11	73,35	7,31	4,18E-02
	Valine	10,13	1,02	14,70	2,18	4,27E-02

A.U.: Arbitrary Units

Supplemental Figure 1: Scheme of treatment (A) and evolution of body weight (B).

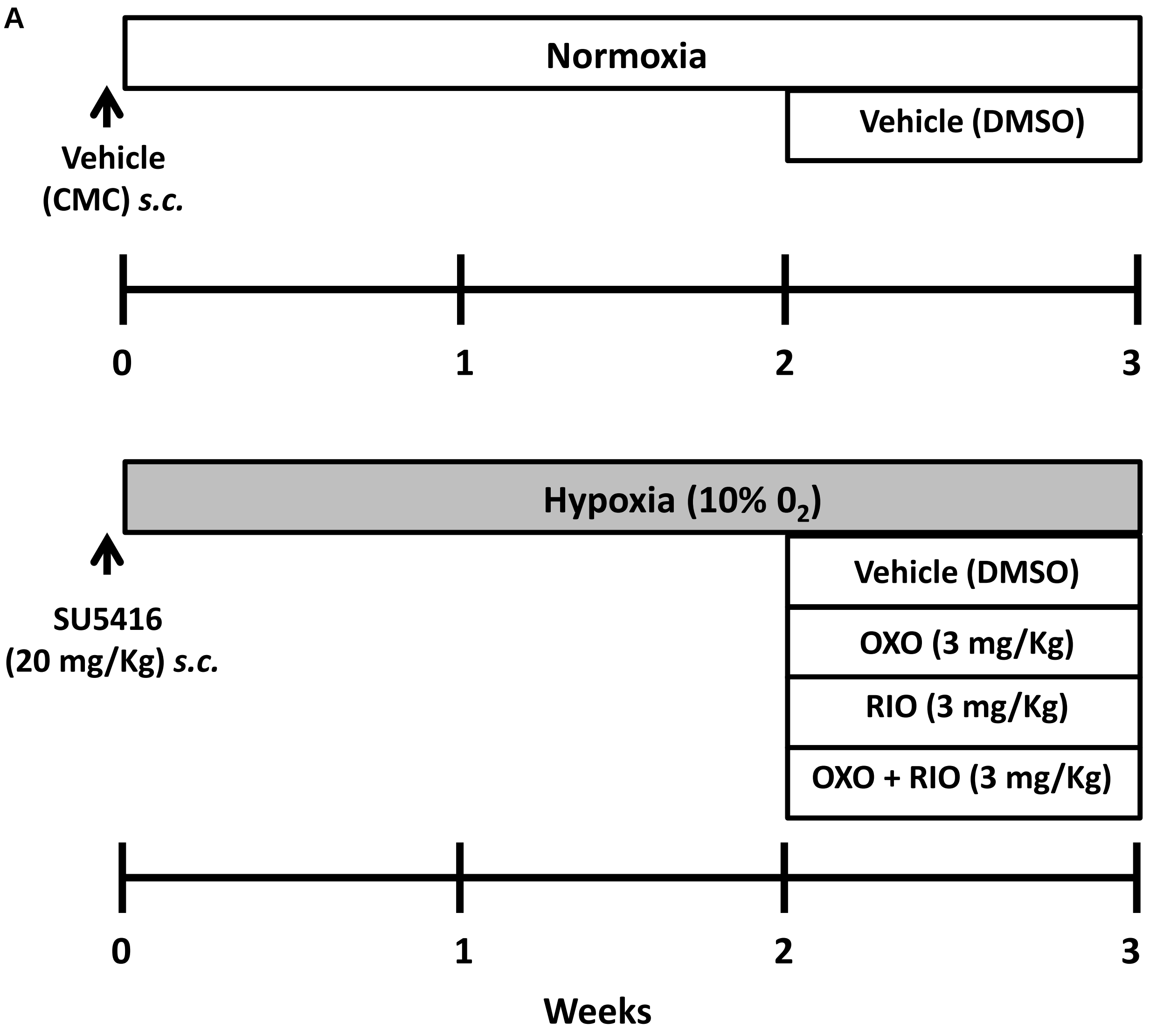

B

	DAY 0			DAY 14			DAY 21		
**NMX-Vh (n=12)**	218.42	±	2.12	283.50	±	6.99	293.58	±	7.65
**SuHyp-Vh (n=10)**	226.70	±	1.84 **	244.90	±	4.74 ***	243.80	±	6.73 ***
**SuHyp-OXO (n=8)**	229.71	±	3.43	241.00	±	6.10 ***	240.57	±	7.86***
**SuHyp-RIO (n=8)**	228.50	±	4.86	235.00	±	10.87***	235.75	±	10.86***
**SuHyp-OXO+RIO (n=8)**	230.38	±	1.22	233.50	±	4.80***	235.88	±	4.13***

Supplemental Figure 2: Principal components analysis (PCA) loading plot highlights the metabolites responsible of the separation between SuHpx and control groups in RV tissue samples.

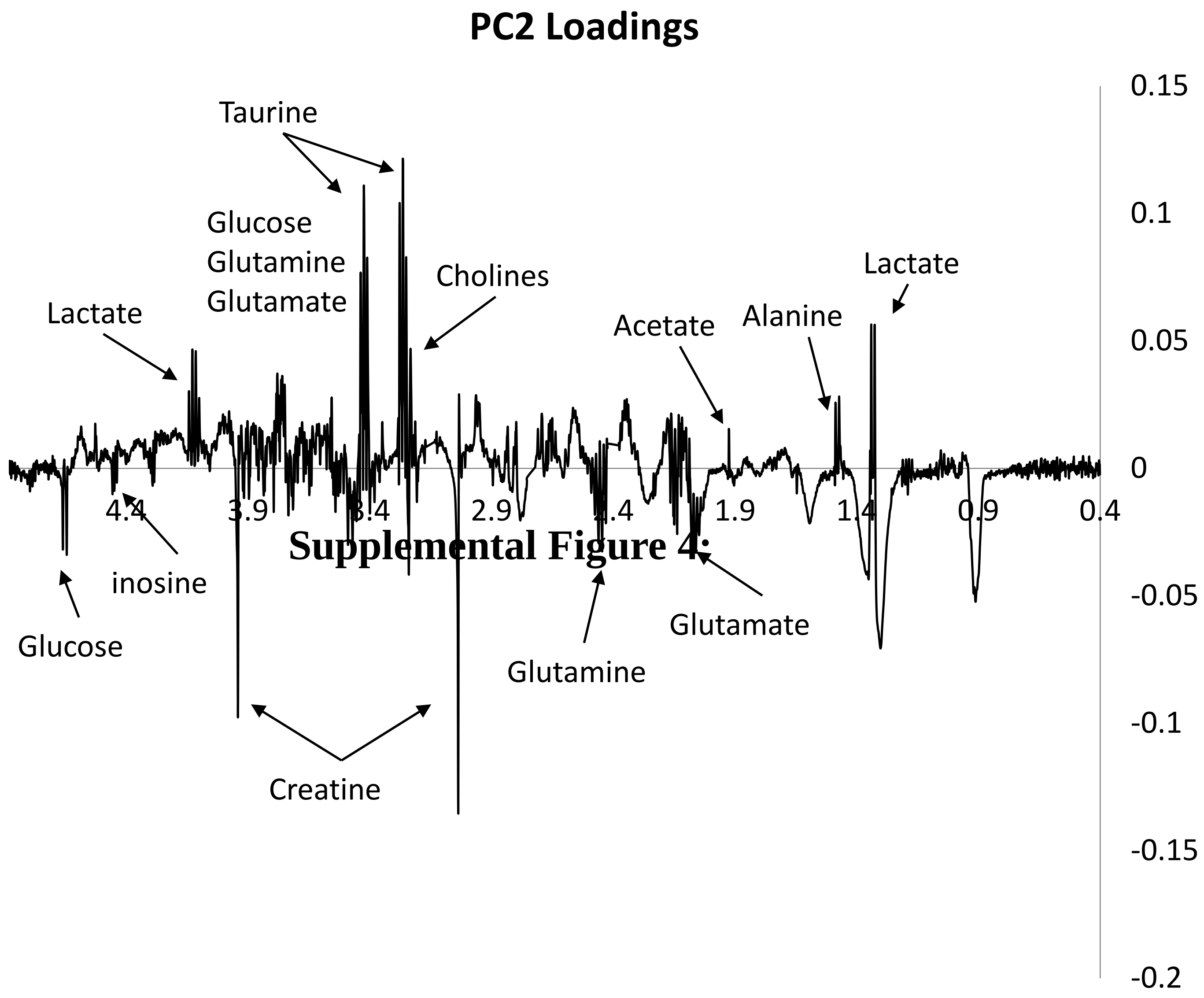

Supplemental Figure 4:

Supplemental Figure 3: Partial Least Square Discriminat Analysis identified the metabolic biomarkers responsible of the separation between **(A)** SuHpx and normoxia group; **(B)** SuHpx and 5(z)-7-oxozeaenol, **(C)** SuHpx and riociguat or **(D)** SuHpx and both drugs combined in right ventricle samples. PLS-DA scores (left) and PLS-DA loadings (right)

A

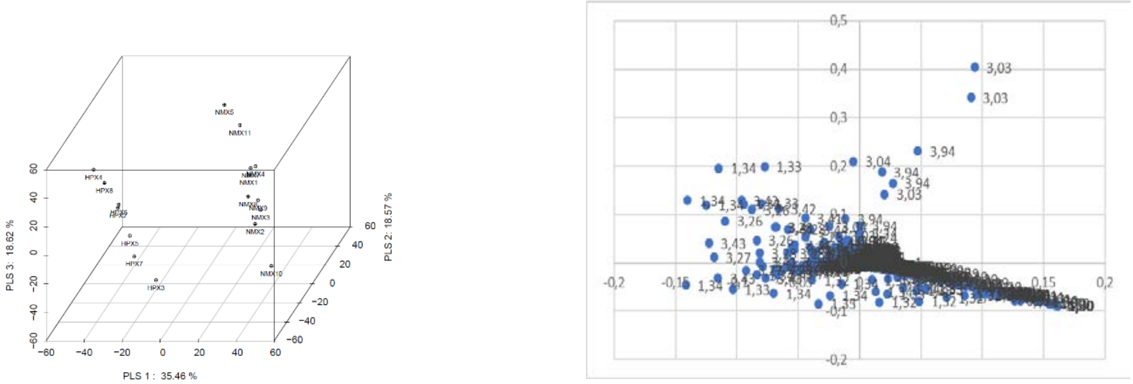

B

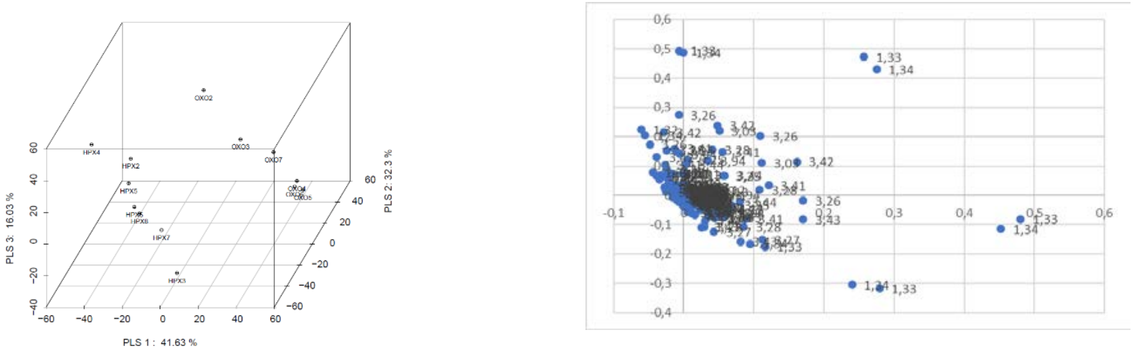

C

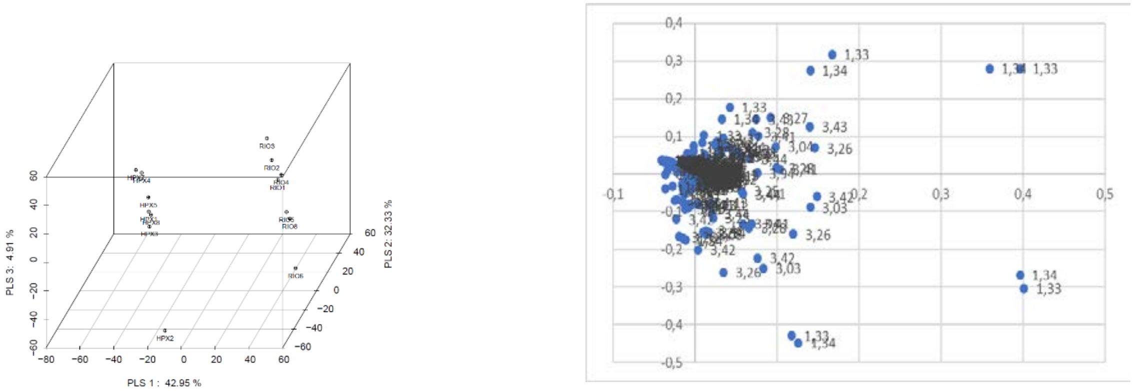

D

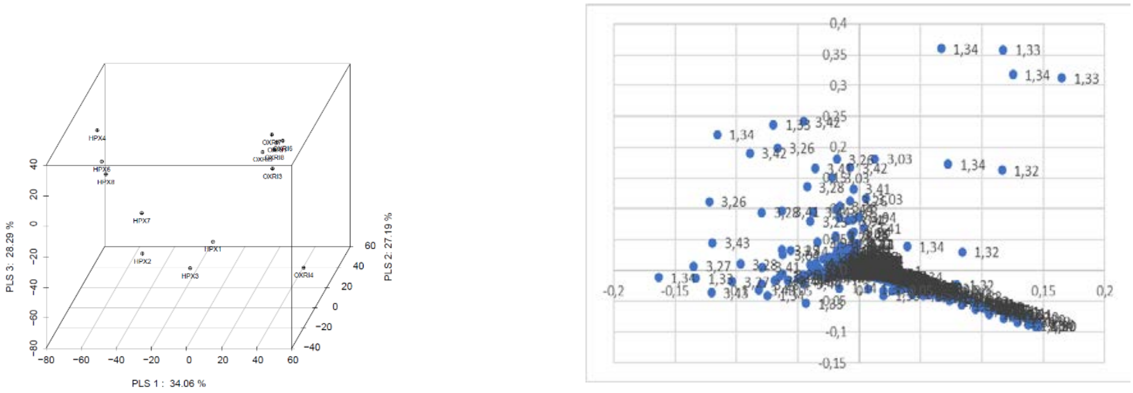

Supplemental Figure 4: Principal components analysis (PCA) loading plot highlights the metabolites responsible of the separation between SuHpx and control groups in lung tissue samples

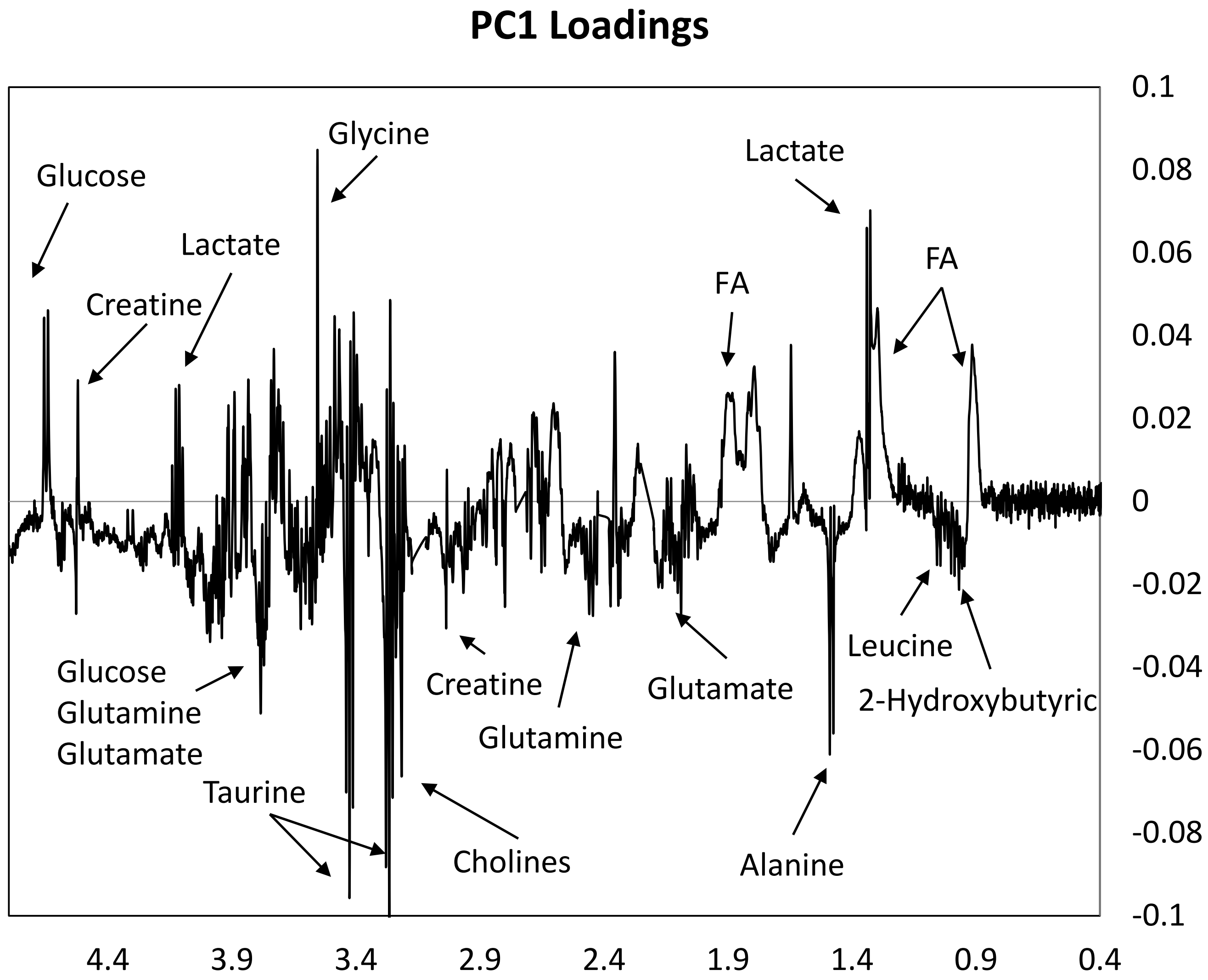

Supplemental Figure 5: Partial Least Square Discriminat Analysis identified the metabolic biomarkers responsible of the separation between **(A)** SuHpx and normoxia group; **(B)** SuHpx and 5(z)-7-oxozeaenol, **(C)** SuHpx and riociguat or **(D)** SuHpx and both drugs combined in lung tissue samples. PLS-DA scores (left) and PLS-DA loadings (right)

A

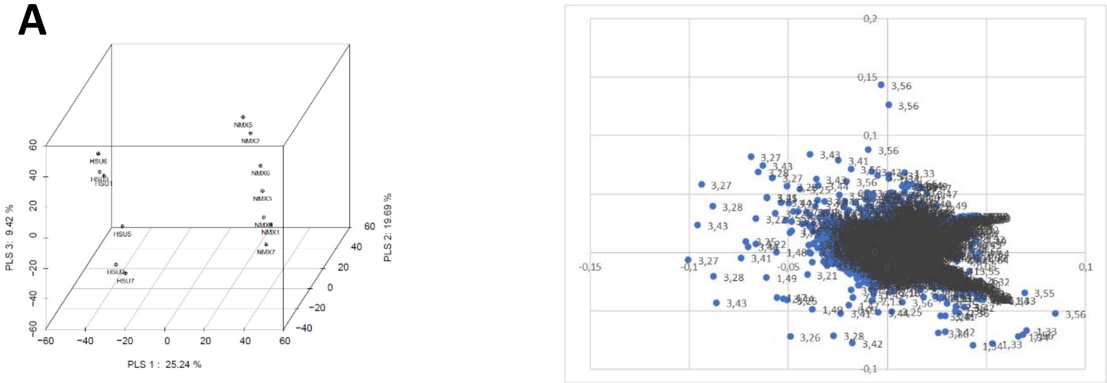

B

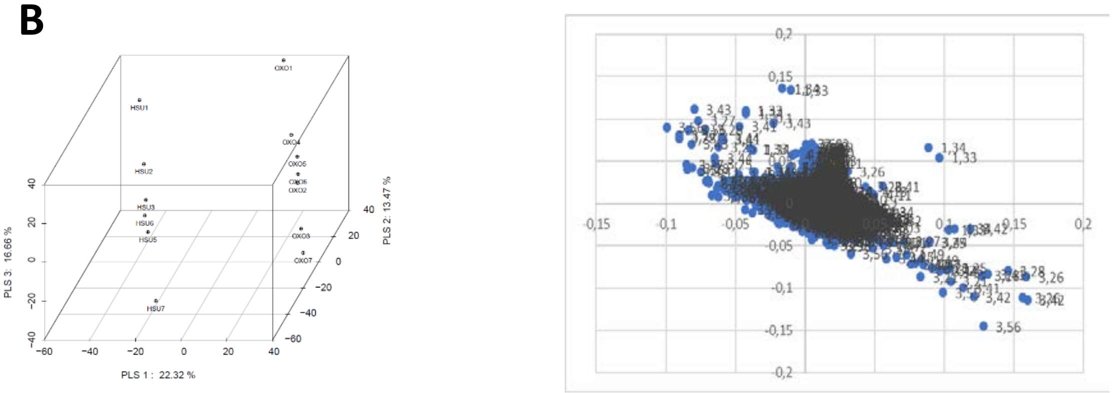

C

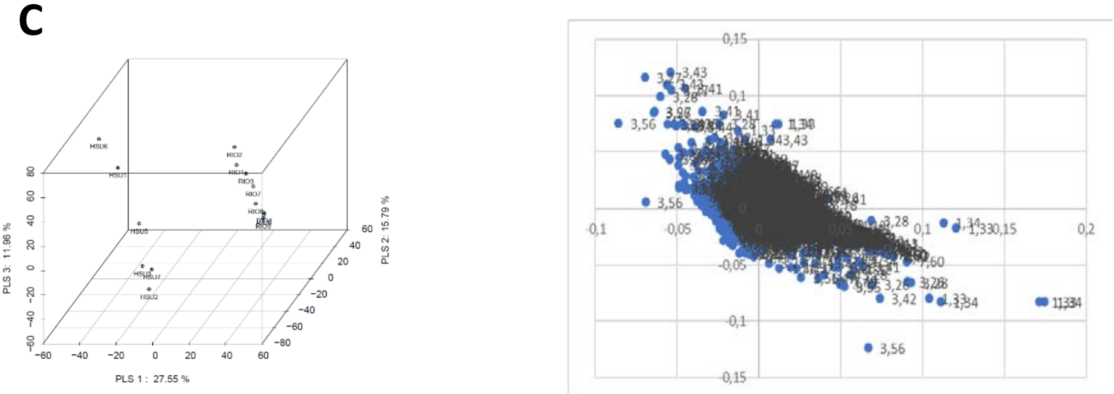

D

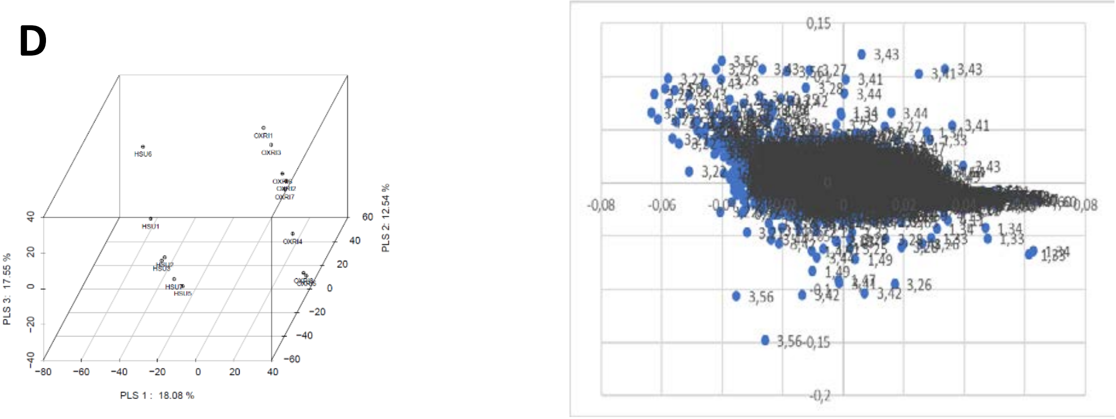

Supplement: Supplementary file 1 [file DataSheet1.PDF]
